# Non-pharmacological Management of Non-productive Chronic Cough in Adults: A Systematic Review

**DOI:** 10.3389/fresc.2022.905257

**Published:** 2022-05-26

**Authors:** Ana Maria Ilicic, Ana Oliveira, Razanne Habash, Yejin Kang, Michelle Kho, Roger Goldstein, Dina Brooks

**Affiliations:** ^1^School of Rehabilitation Science, McMaster University, Hamilton, ON, Canada; ^2^West Park Healthcare Centre, Toronto, ON, Canada; ^3^Lab 3R Respiratory Research and Rehabilitation Laboratory, University of Aveiro (ESSUA), Aveiro, Portugal; ^4^Department of Medical Sciences, iBiMED – Institute of Biomedicine, University of Aveiro, Aveiro, Portugal; ^5^Faculty of Health Sciences, McMaster University, Hamilton, ON, Canada; ^6^St. Joseph's Healthcare, Hamilton, ON, Canada; ^7^The Research Institute of St. Joe's, St. Joseph's Healthcare, Hamilton, ON, Canada; ^8^Department of Physical Therapy, University of Toronto, Toronto, ON, Canada

**Keywords:** chronic cough, dry cough, respiratory diseases, non-pharmacological therapy, alternative therapy

## Abstract

**Background:**

Chronic cough is a common reason for medical referral and its prevalence is on the rise. With only one pharmaceutical therapy currently under review for the treatment of refractory chronic cough, exploring non-pharmacological chronic cough management therapies is important. This systematic review summarizes the effectiveness of non-pharmacological chronic cough therapies in adults with non-productive refractory chronic cough or cough due to chronic respiratory diseases.

**Methods:**

We searched Medline, Embase, Cochrane, CINAHL, and Scopus from inception to September 2021. Randomized controlled trials published in English, Portuguese, or French, and examining the effects of non-pharmacological therapies in adults with chronic non-productive cough (>8 weeks; <2 teaspoons sputum) were included. Mean differences, medians, and odds ratios were calculated as appropriate.

**Results:**

16,546 articles were identified and six articles representing five unique studies were included. Studies evaluated 228 individuals with refractory chronic cough or chronic cough due to a chronic respiratory disease [162 women (71%); 52 ± 11 to 61 ± 8 years old]. Obstructive sleep apnea was the only chronic respiratory disease studied. Non-pharmacological therapies included education, cough suppression, breathing techniques, mindfulness, and continuous positive airway pressure. When standing alone, non-pharmacological cough therapies improved cough-specific health related quality of life when not associated with interventions (mean diff MD 1.53 to 4.54), cough frequency (MD 0.59 95%CI 0.36 to 0.95), and voice outcomes (MD 0.3 to 1) when compared to control interventions.

**Conclusion:**

The evidence of non-pharmacological therapies for non-productive chronic cough is limited. Existing studies reflect the heterogeneity in study design, sample size, and outcome measures. Thus, clinical recommendations for using the most effective interventions remain to be confirmed.

## Introduction

Cough is one of the body's most important reflexes, acting as a primary defense mechanism to clear the upper airways. When cough becomes chronic, defined as lasting for 8 weeks or more (1), it can drastically impair activities of daily living and health-related quality of life (1, 2), contributing to a downward spiral of fatigue, embarrassment, frustration, anxiety, depression, and social isolation (3, 4). These negative psychosocial impacts are aggravated by the stigma associated with coughing, especially during the recent covid-19 pandemic (5).

The global prevalence of chronic cough in otherwise healthy individuals is increasing, with a prevalence of 16–18% in Canada (2), 18% in the USA (1), and 33% in Europe (1). In people with chronic respiratory diseases, its prevalence has been reported to be 30–90% (6). Chronic cough is also one of the most common reasons for medical referrals (2). Costs associated with chronic cough correspond, on average, to $3,266 per patient, which includes multiple medical appointments, prescription medications, and hospitalizations (7). Despite medical management, patients often report minimal or no improvement in their chronic cough and turn to over-the-counter medications, at an estimated annual cost of $1–3.5 billion for temporary symptom relief (7). Thus, treating chronic cough has become a priority, both, among otherwise healthy individuals and those with underlying chronic respiratory diseases (6, 8).

The two main forms of chronic cough management are pharmacological and non-pharmacological therapy (9–11). Currently there is only one pharmaceutical therapy under review for the treatment of refractory chronic cough (12), but approval is pending. Other commonly used pharmacological therapies include antacids, pro-motility agents, and neuromodulators (6), however, their effectiveness is limited and may be associated with significant adverse effects such as dizziness, fatigue, cognitive changes, nausea, and risk of withdrawal (5). Non-pharmacological therapies include, but are not limited to, education, cough suppression, and breathing techniques. They have been reported to be equally effective, with fewer side effects, compared to pharmacological therapies (6, 13), however, there is a paucity of information regarding which non-pharmacological therapies are most effective. Four systematic reviews of non-pharmacological management of chronic cough have been published in the past decade (9, 14–16), however they focused mainly on speech language pathology, neglecting other therapies such as behavioral therapies or relaxation (14, 16), or focused on people with only refractory chronic cough, excluding those with chronic cough due to chronic respiratory diseases (9, 15). Our systematic review adds to this field of research by including, both, people with refractory chronic cough or chronic respiratory diseases, and seeking to identify all non-pharmacological therapies. This updated systematic review will help guide healthcare providers in the implementation of effective cough management therapies for individuals with either refractory chronic cough or chronic respiratory diseases.

The primary objective of this systematic review is to summarize the effects of non-pharmacological cough management strategies on cough-related quality of life in adults with non-productive refractory chronic cough or with an underlying chronic respiratory disease. The secondary objectives are to summarize the characteristics of individuals participating in non-pharmacological cough management strategies, the structure and components of different cough management strategies reported in the literature, and the effects of cough management strategies on health-specific and cough-related outcomes.

## Methods

This systematic review was conducted according to the Cochrane Handbook for Systematic Reviews of Interventions and reported following the Preferred Reporting Items for Systematic Reviews and Meta-Analysis (PRISMA) guidelines (17, 18) (Supplementary Material A). The protocol was registered with the International Prospective Register of Systematic Reviews network (no. CRD42020200015) and approved on August 21st, 2020.

### Literature Search

Prior to conducting a search, a librarian was consulted to determine effective search strategies. Two authors (A.O. and A.M.I) conducted a search of the following databases: Medline, Embase, Cochrane, Cumulative Index of Nursing and Allied Health Literature (CINAHL), and Scopus from inception to September 2020. The search was updated in September 2021. For each database, a search utilizing both keywords and medical subject heading (MESH), designed to identify all non-pharmacological cough interventions for people with chronic cough, was performed within the titles and abstracts of records. An example of the search strategy, conducted in MEDLINE, is reported in Supplementary Material B.

### Eligibility Criteria

Articles were deemed eligible if the following criteria was met: (1) randomized controlled trials (RCTs); (2) included adults (≥18 years) with refractory chronic cough (>8 weeks) or those with underlying chronic lung diseases, (3) reported minimum to no sputum production (i.e., <2 teaspoons/day); (4) examining the effectiveness of non-pharmacological therapies alone (e.g., cough education, laryngeal irritation reduction strategies, cough control, psychoeducational strategies or behavioral therapies), and were (5) written in English, Portuguese, or French. Articles were excluded if: (1) patients presented with an acute respiratory condition (cough <8 weeks); (2) the duration of cough was not defined; (3) interventions included pharmaceuticals, dietary supplements, or surgery; (4) invasive non-pharmacological interventions (e.g., acupuncture), (5) abstracts in conference proceedings, systematic reviews, dissertations, editorials, case reports, or book chapters. All articles were included independently of the outcome assessed, except for capsaicin and citric acid cough challenge, which were excluded as these tests are used to study mechanisms of disease rather than efficacy of the specified cough therapies (19).

### Study Selection

Citations were first managed in EndNote X8.2 (Clarivate, Philadelphia, Pennsylvania, USA) for duplicates screening and removal and were then uploaded to Covidence (Covidence, Boston, Massachusetts, USA) for the study selection process. Four independent reviewers worked in pairs (A.O., A.M.I., R.H., and Y.K.) to screen the titles and abstracts: consensus between at least two reviewers was needed before a final decision to include or exclude the study was made. Remaining article full texts were then independently screened by two reviewers (A.O. and A.M.I.). All disagreements were resolved via consensus and a third reviewer was consulted (D.B.) if a consensus could not be reached.

### Data Extraction

Data from the eligible articles was extracted using a data extraction form, designed prior to data collection, which included information regarding study characteristics, program characteristics, and results. Article characteristics included the first author's last name, year of publication, country of origin, experimental and control interventions, follow-up period duration, drop-out rates at any point in the study, participant's comorbidities, and demographics (i.e., total number of participants, age, and gender) per experimental and control groups. When dropout rates were not reported in the articles, they were calculated as (total randomized—total completed the study protocol)/total randomized) ^*^ 100. Program characteristics included the duration and frequency of the intervention, equipment used, inclusion and exclusion criteria, outcomes and outcome measures, and results. Data extraction was pilot tested by two reviewers (A.O. and A.M.I.) in one study to clarify any discrepancies. Data from the remaining articles were extracted by one reviewer (A.M.I.) and verified by a second reviewer (A.O.).

### Risk of Bias Assessment

Risk of bias assessments were conducted using the Revised Cochrane risk-of-bias tool for randomized trials (RoB) (17, 20), which evaluates 5 domains: randomization process, deviations from intended interventions, missing outcome data, measurement of the outcome, and selection of the reported result (1). Authors (A.O. and A.M.I.) piloted the risk of bias assessment on one article and then conducted the assessment for the remaining articles individually. Disagreements were solved by consensus.

### Data Analysis

A meta-analysis was planned to be conducted if the articles were similar enough to be grouped together (i.e., present with similar interventions, populations, and outcomes). When a meta-analysis was not possible to conduct, the median and interquartile ranges, mean differences (MD) and 95% confidence intervals (95%CI), or odds ratios were extracted directly from the studies or calculated using Review Manager 5.4.1, according to the Cochrane Handbook for Systematic Reviews of Interventions (18).

## Results

### Literature Search and Study Selection

**Figure 1:**
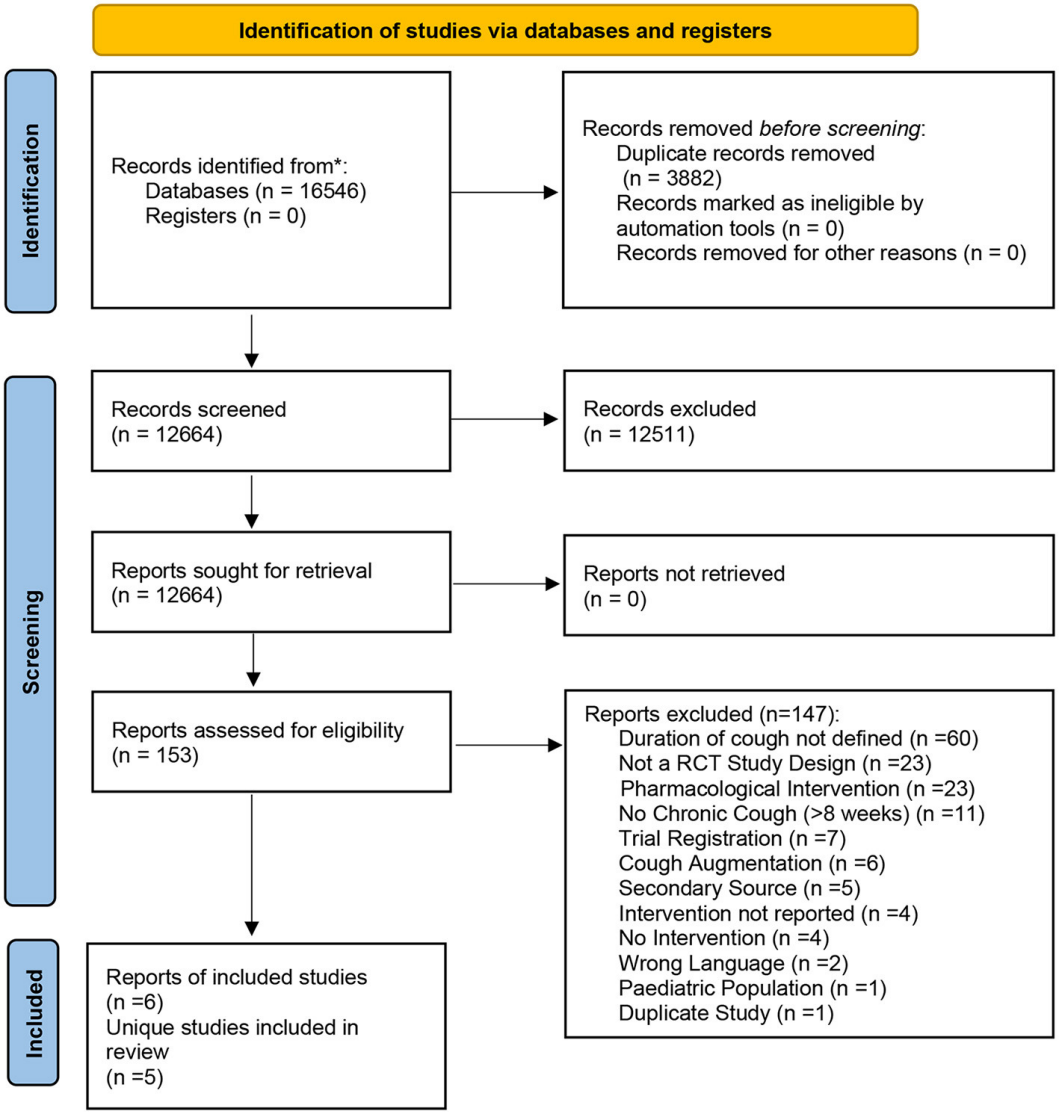
Flowchart of records and studies included in the systematic review.

The database search identified 16,546 records. After duplicate removal (*n* = 3,882), 12,664 records underwent title and abstract screening and 153 were identified for full-text screening. At this stage, 147 records were excluded for not meeting the eligibility criteria (Supplementary Material C). This yielded a total of six records [five unique studies—(21, 22) analyzed the same sample of participants and thus were counted as one unique study] included in this review (21–26). The PRISMA flowchart of the study selection process is provided in Figure 1. A meta-analysis was not possible to conduct due to heterogeneity of study populations, interventions, and outcomes used.

### Study Characteristics

**Figure 2:**
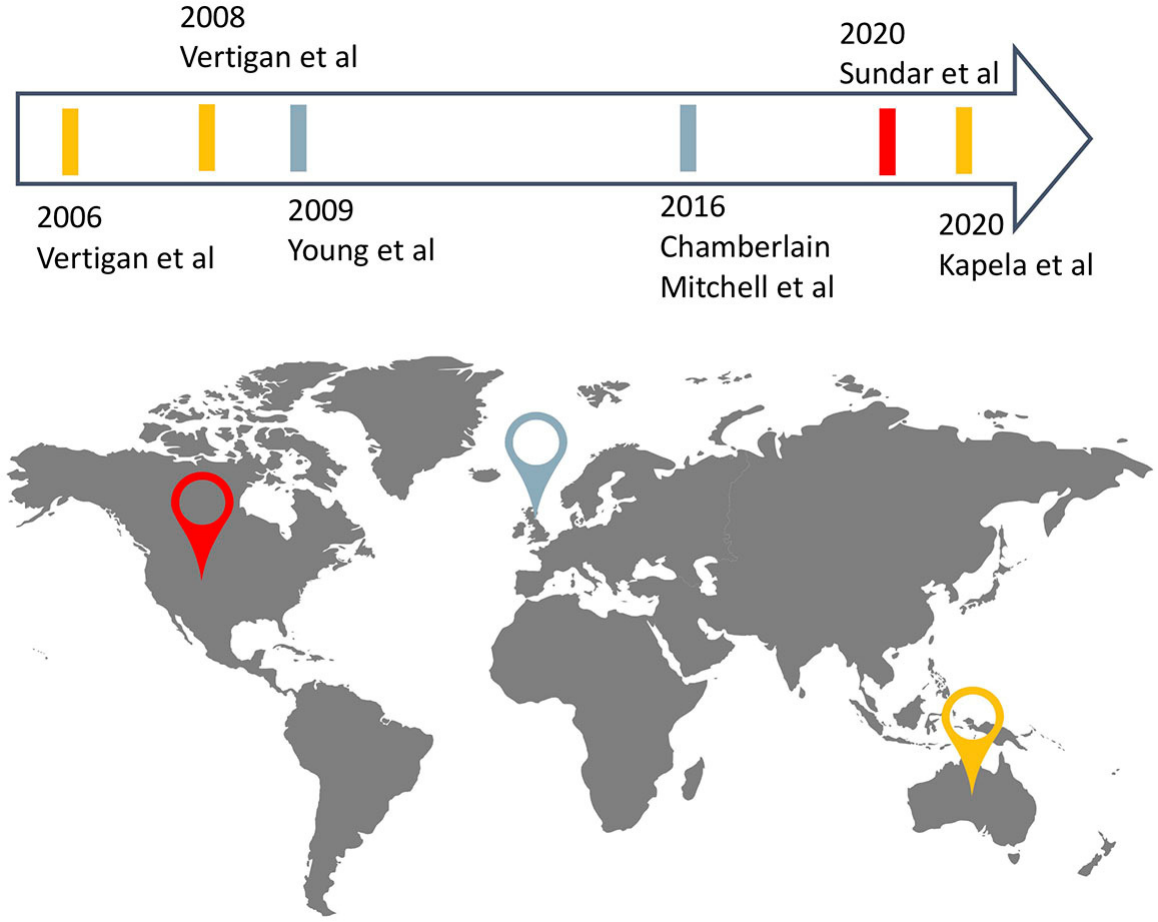
Countries of origin and date of publication of records included in the review.

Included articles were published between 2006 and 2020, and studies took place in Australia (*n* = 2), the United-Kingdom (*n* = 2), and the United States of America (*n* = 1). One article reported on a multicentre study (24) and the remaining five, on single-center studies (21–23, 25, 26), totalling 228 participants (114 in the experimental groups, 114 in the control group), among the five studies (Figure 2). Sample sizes ranged from 9 to 43 with dropouts ranging from 0% to 35% in experimental groups and 0 to 34% in control groups. A detailed description of study characteristics is presented in Table 1.

**Table 1:** Study Characteristics.

				**Experimental group**				**Control group**			
**References**	**Country**	**Interventions**	**Co-morbidities**	***n***	**Drop-out rates (%)**	**Age (years)**	**Sex (M) (*N*, %)**	***n***	**Drop-out rates (%)**	**Age (years)**	**Sex (M) (*N*, %)**
Vertigan et al. (21)*	Australia	Experimental: SPEICH-C Control: healthy lifestyle education	Asthma PNDS GERD PVFM	43	8.5	57.5 ± 13.8	8, 19	44	12	61.3 ± 13.2	15, 34
Vertigan et al. (22)*				40	14.8	58.9 ± 13.6	7, 18	43	16	61.5 ± 13.3	15, 35
Young et al. (23)	UK	Experimental 1: Mindfulness Experimental 2: Cough Suppression Control: No intervention	Asthma GERD UACS	Cough Suppression: 9 Mindfulness: 10	0	Cough Suppression 61.1 ± 8.4 Mindfulness: 60.2 ± 8.1	Cough Suppression 4, 44 Mindfulness: 3, 30	11	0	54.2 ± 10.8	3, 27
Chamberlain et al. (24)	UK	Experimental: PSALTI Control: Healthy Lifestyle Advice	N/A	34	35	61 [53-67]	9, 26	41	34	56 [48-67]	15, 37
Sundar et al. (25)	USA	Experimental: CPAP Control: Sham CPAP	OSA	9	25	52.4 ± 10.9	2, 22	9	10	62.7 ± 6.3	5, 56
Kapela et al. (26)	Australia	Experimental: SPEICH-C + breathing videos Control: SPEICH-C	GERD Rhinosinusitis Asthma ACE inhibitor withdrawal	9	11	59 ± 17	0, 0	9	11	57 ± 9	2, 22

ACE, Angiotensin Converting Enzyme; CPAP, Continuous Positive Airway Pressure; GERD, Gastroesophageal Reflux Disease; OSA; Obstructive Sleep Apnea; PSALTI, Physiotherapy, Speech and Language Therapy Intervention; PNDS, Postnasal Drip Syndrome; PVFM, Paradoxical Vocal Fold Movement; SLP, Speech-language Pathology; SPEICH-C, Speech Pathology Evaluation, and Intervention for Chronic Cough; UACS, Upper Airway Cough Syndrome; UK, United Kingdom; USA; United States of America. ^*^Vertigan et al. published two separate studies, however using the same population for both. For our review, study table summaries are provided together due to the nature of the article origin. ^*^Participant totals were calculated using the highest participant number in either Vertigan et al. article. As the participant population was derived from the same data, study numbers were only counted once.

Eligibility criteria was comparable across the majority of the included articles and required that participants have a cough lasting for 8 weeks or more (21–26), have a refractory chronic cough (failed treatment for other possible causes of cough such as asthma, COPD, gastro-esophageal reflux disease (GERD), rhinitis) (21, 22, 24, 26) or a chronic cough from an associated chronic respiratory disease (OSA) (25), and had normal chest imaging (21, 22, 24, 25). Articles excluded participants if there was history of a recent upper respiratory tract infection in the past 4–6 weeks (21–26).

In both experimental and control groups, participants were mainly women (experimental: *n* = 88; 77%; control: *n* = 74; 65%) with ages ranging from 52 ± 11 to 61 ± 8 years old in experimental groups and 54 ± 11 to 63 ± 6 years in control groups. Comorbidities in both groups included GERD (21–23, 26), asthma (21–23, 26), upper airway cough syndrome (UACS) (23), angiotensin converting enzyme (ACE) inhibitor withdrawal (21, 22, 26), postnasal drip syndrome (PNDS) (21, 22), paradoxical vocal fold movement (PVFM) (21, 22), OSA (25), and rhinitis (26).

### Intervention Characteristics

Duration of interventions ranged from 1 to 8 weeks, with two articles not reporting the length of the intervention (22, 26). The experimental group interventions included mindfulness (23), cough suppression (23), continuous positive airway pressure (CPAP) therapy (25), education strategies to reduce cough, laryngeal hygiene and hydration strategies, cough control, and psychoeducational counseling delivered through speech-language therapy (21, 22), speech language pathology with video breathing exercises (26), and through physiotherapy and speech and language therapy (PSALTI) (24). The control interventions were healthy lifestyle education and advice (19, 20, 22), sham CPAP therapy (25), strategies to reduce cough, laryngeal hygiene, hydration, cough control, and psychoeducational counseling by a speech language pathologist (SLP) without breathing exercises (26) or no intervention (23). A detailed description of interventions characteristics is presented in Table 2.

**Table 2:** Intervention Characteristics.

**References**	**Cough therapy**	**Components**	**Duration & frequency**	**Equipment**	**Inclusion criteria**	**Exclusion criteria**
Vertigan et al. (21) Vertigan et al. (22)	SPEICH-C	1) Education 2) Cough control 3) Psycho-educational counseling 4) Vocal Hygiene Education	8 weeks 4 sessions: 30 min each	N/A	1) >18 years; 2) Ability to attend the sessions; 3) Cough >8 weeks despite optimal medical treatment 4) sought medical attention	1) recent URI; 2) Untreated underling condition; 3) abnormal chest X-ray; 4) COPD; 5) neurological voice disorder
Young et al. (23)	*Two groups:* 1) Mindfulness 2) Voluntary Suppression	Mindfulness: Controlled breathing and Meditation Cough suppression: Voluntary	1 week Mindfulness: 30 min/day then 15min/day training exercise prior to second cough challenge. Cough suppression: performed during the challenge	Mindfulness: audiocassette for home practice	1) Cough >8 weeks despite optimal medical treatment; 2) Referral to a cough clinic	1) Did not have a measurable C5; 2) URI in past 4 weeks; 3) Current treatment with opiates, ACE inhibitors, OTC cough medicine; 4) Current smokers
Chamberlain et al. (24)	PSALTI:	1)education; 2) laryngeal hygiene and hydration; 3) cough suppression techniques; 4) breathing exercises; 5) psychoeducational counseling	4 weeks 4 sessions: 45 min each	N/A	1) Older than 18 years; 2) Chronic cough (>8 weeks) despite optimal medical treatment for underlying conditions; 3) Normal Chest x-ray; 4) <10 mL sputum/day	1) URI within 4 weeks; 2) ACE inhibitors; 3) Current smokers; 4) Respiratory disease; 5) Vocal cord nodules, malignancy, or active aspiration
Sundar et al., 2020 (25)	CPAP Therapy	CPAP equipment provided by Philips-Respironics Inc.	6 weeks	CPAP equipment provided by Philips-Respironics Inc	1) Older than 18 years; 2) Chronic cough (>8 weeks) despite optimal medical treatment for underlying conditions; 3) Smoking <5 pack years and a history of more than 10 years; 4) Normal chest imagology tests; 6) FEV1/FVC > 0.7, FVC > 70% predicted and DLCO>50% predicted; 7) Diagnosis of OSA	1) Pregnancy; 2) Positive methacholine challenge test; 3) Asthma; 4) Pneumonia <6 months; 5) Congestive heart failure, renal disease, liver disease, pulmonary embolism, stroke or neurodegenerative disease, malignancy; 6) > 70 years; 7) Use of supplemental oxygen or CPAP; 8) Opiates, benzodiazepines; 9) Alcoholism, drug dependence or illicit drug use; 10) Prior GI or laryngeal surgery; 11) Craniofacial abnormalities that preclude CPAP placement.
Kapela et al. (26)	SPEICH-C + pre-recorded SPEICH-C technique videos	SPEICH-C Component: 1) Education 2) Reduce laryngeal irritation 3) Cough suppression strategies 4) Psycho-educational counseling SPEICH-C technique videos: Videos demonstrating therapy exercises	1 to 6 sessions	Computer or DVD player	1) Older than 18 years old; 2) access to a computer of DVD player; 3) Chronic cough (>8 weeks) despite optimal medical treatment for underlying conditions; 4) sought medical attention due to cough	1) Current smoker or ceased smoking less than 6 weeks prior to enrollment; 2) Recent upper respiratory tract infection; 3) Cognitive disorders precluding participation; 4) Untreated associated conditions including asthma, rhinosinusitis, gastro-oesophageal reflux disease, use of angiotensin-converting-enzyme inhibitors, or lung disease.

*ACE, Angiotensin Converting Enzyme; COPD, Chronic Obstructive Pulmonary Disease; CPAP, Continuous Positive Airway Pressure; FEV1, Forced expiratory volume in 1 second; FVC, Forced Vital Capacity; DLCO, Diffusing Capacity of the Lung for Carbon Monoxide; GERD, Gastroesophageal Reflux Disease; OSA; Obstructive Sleep Apnea; OTC, Over the counter; PSALTI, Physiotherapy, Speech and Language Therapy Intervention; PNDS, Postnasal Drip Syndrome; SPEICH-C, Speech Pathology Evaluation and Intervention for Chronic Cough; UACS, Upper Airway Cough Syndrome; X-Ray, Energetic High-Frequency Electromagnetic Radiation*.

### Effects of Interventions

Out of the six included articles, three reported the effects of interventions on cough-related quality of life (primary outcome) (24–26), one reported on general and disease-specific health-related quality of life (23), two on cough frequency (objective cough counts) (23, 24), four articles reported on symptoms [i.e., breathing (21), cough (21), upper airway symptoms (21), urge to cough (23), cough severity (24, 26), anxiety and depression (23, 24)], and five articles reported on other outcomes such as sinonasal disease, markers of airway inflammation, and voice (21, 22, 24–26). In total, seventeen outcome measures were used to evaluate cough interventions, with the Leicester Cough Questionnaire (LCQ) most commonly used in three articles (24–26). Outcome scores were collected at baseline (21–26), and 1 week (23), 4 weeks (24), 6 weeks (25), 8 weeks (21, 22) and 3-months after baseline (24, 25). One article reported outcomes after a number of sessions (1–6) rather than a fixed time (26). A detailed description of the effects of interventions can be found in Table 3.

**Table 3:** Result Characteristics.

**References**	**Interventions**	**Outcomes**	**Outcome measures**	**Results**	**Summary of findings**
Vertigan et al. (21)	Experimental: SPEICH-C Control: Equivalent course of healthy lifestyle education	1) Symptoms: breathing; cough; voice; upper airway, limitations 2) limitation of symptoms on everyday activity clinical judgement	**1) Symptom rating 5-point scale** a) Total score b) Breathing c) Voice d) Upper airway e) Limitations 2) Clinical Judgment (Successful vs. Partially successful vs. Unsuccessful)	1) Symptoms (Pre/post scores): - Total score—EG: 35.4 ± 16 vs. 22.7 ± 18; CG: 29.9 ± 13.5 vs. 28.8 ± 16.5 (*p < * 0.001) - Breathing—EG: 7.9 ± 4.1 vs. 5 ± 4.2; CG: 6.6 ± 4.7 vs. 5.5 ± 3.5 (*p < * 0.001) - Cough—EG: 8.8 ± 2.8 vs. 4.9 ± 3; CG: 7.5 ± 3.6 vs. 6.3 ± 3.5 (*p =* 0.003) - Voice—EG: 7.2 ± 6 vs. 4.7 ± 5.2; CG: 6.5 ± 4.6 vs. 6.2 ± 5 (*p =* 0.005) - Upper airway—EG: 9.2 ± 6.6 vs. 6.5 ± 6.3; CG: 7.4 ± 4.9 vs. 7.4 ± 5.5 (*p =* 0.002) - Limitations—EG: 2.3 ± 1.2 vs. 1.6 ± 1; CG: 2.2 ± 1.1 vs. 2 ± 1 (*p =* 0.011) 2) Clinical Judgment (Successful vs. Partially successful vs. Unsuccessful) EG: 38 vs. 3 vs. 2; CG: 6 vs. 3 vs. 35 (*p < * 0.001)	SPEICH-C resulted in better outcomes on the 5-point symptom rating scale and on the clinical judgement scores in comparison to the control group.
Vertigan et al. (22)	Experimental: SPEICH-C Control: Equivalent course of healthy lifestyle education	1) Perceptual Voice Outcomes 2) Acoustic outcomes and Electroglottography	**1) Ratings of the reading the grandfather passage** 2) Praat acoustics analysis program and laryngograph Speech Studio, Laryngograph	1) Reading the grandfather passage (Pre/post scores) - High Pitch—EG: 1.0 ± 0.2 vs. 1.1 ± 0.5; CG: 1.1 ± 0.2 vs. 1.0 ± 0.2 (*p =* 0.273) - Low Pitch—EG: 1.2 ± 0.5 vs. 1.1 ± 0.4; CG: 1.4 ± 0.6 vs. 1.3 ± 0.7 (*p =* 0.899) - Monotone—EG: 1.3 ± 0.5 vs. 1.2 ± 0.6; CG: 1.2 ± 0.5 vs. 1.2 ± 0.4 (*p =* 0.777) - Soft—EG: 1.3 ± 0.8 vs. 1.1 ± 0.5; CG: 1.3 ± 0.6 vs. 1.1 ± 0.4 (*p =* 0.902) - Loud—EG: 1.0 ± 0.2 vs. 1.0 ± 0.0; CG: 1.0 ± 0.0 vs. 1.0 ± 0.0 (*p =* 0.344) - Breathy—EG: 2.4 ± 1.2 vs. 1.5 ± 0.9; CG:. 2.4 ± 1.2 vs. 2.4 ± 1.0 (*p < * 0.001) - Strain—EG: 2.7 ± 1.3 vs. 1.9 ± 1.1; CG: 2.6 ± 1.0 vs. 2.6 ± 1.0 (*p < * 0.001) - Rough—EG: 2.7 ± 1.2 vs. 1.9 ± 1.2; CG: 2.6 ± 1.1 vs. 2.8 ± 1.1 (*p < * 0.001) - Glottal Fry—EG: 2.1 ± 1.2 vs. 1.3 ± 0.7; CG: 2.0 ± 1.2 vs. 2.1 ± 1.1 (*p =* 0.001) - Pitch Breaks—EG: 1.1 ± 0.5 vs. 1.1 ± 0.0; CG: 1.1 ± 0.3 vs. 1.1 ± 0.2 (*p =* 0.478) - Phonation breaks—EG: Pre 1.1 ± 0.6 vs. Post 1.0 ± 0.0; CG: Pre 1.1 ± 0.3 vs. Post 1.0 ± 0.2 (*p =* 0.439) - Voice arrests—EG: Pre 1.1 ± 0.6 vs. Post 1.0 ± 0.0; CG: Pre 1.0 ± 0.0 vs. Post 1.1 ± 0.4 (*p =* 0.042) - Falsetto—EG: Pre 1.0 ± 0.2 vs. Post 1.0 ± 0.0; CG: Pre 1.0 ± 0.0 vs. Post 1.0 ± 0.0 (*p =* 0.344) 2) Praat acoustics analysis program and Laryngograph (Pre/post scores) - MPT—EG: 9.4 ± 6.4 vs. 11.0 ± 5.6; CG: 10.8 ± 6.4 vs. 11.6 ± 6.6 (*p =* 0.422) - SDF—EG: 18.6 ± 12.3 vs. 17.7 ± 14.2; CG: 25.0 ± 16.2 vs. 23.7 ± 17.3 (*p =* 0.970) - Jitter—EG: 2.6 ± 2.5 vs. 1.6 ± 1.3; CG: 2.4 ± 1.6 vs. 2.1 ± 1.5 (*p =* 0.209) - HNR—EG: 17.1 ± 5.9 vs. 19.7 ± 5.0; CG: 19.0 ± 5.1 vs. 18.6 ± 5.5 (*p =* 0.200) - DFx (male)—EG: 97.3 ± 13.1 vs. 96.7 ± 12.3; CG: 105.7 ± 16.6 vs. Post 103.0 ± 16.3 (*p =* 0.746) - DFx (female)—EG: 167.4 ± 27.1 vs. 167.7 ± 21.6; CG: 178.3 ± 29.8 vs. 177.1 ± 32.0 (*p =* 0.801) -Qx—EG: 39.3 ± 17.9 vs. 43.3 ± 19.5; CG: 33.2 ± 16.6 vs. 37.2 ± 19.0 (*p =* 0.449)	SPEICH-C resulted in better voice outcomes breathing, strain, and rough scores on perceptual in comparison to the control group. No significant differences between groups were observed for changes in acoustic and electroglottography outcomes.
Young et al. (23)	Experimental 1: Mindfulness Experimental 2: Cough Suppression Control: No intervention	1) Urge to cough	1) Modified Borg Scale	1) Modified Borg scale (Pre/post mean differences) - Urge to cough - Voluntary suppression 0.0 (−1.0 to 1.0); mindfulness 0.0 (0.0 to 2.0); CG: 0.0 (−2.0 to 1.0); (*p =* 0.7)	No significant differences between groups were observed for changes in urge to cough.
Chamberlain et al. (24)	Experimental: PSALTI Control: Healthy Lifestyle Advice	**1) Cough- related QoL** 2) Objective Cough frequency 3) Cough severity	**1) LCQ** 2) LCM 3) VAS 4) VPQ 5) SF-36 6) HADS	Pre/post mean differences 1) LCQ Total score—EG: 3.40 (2.26 to 4.55); CG: 1.66 (0.78 to 2.54) (*p =* 0.024) 2) LCM (Cf/hr)—EG: Mean diff 0.55 (0.33 to 0.75); CG: 0.82 (0.60 to 1.22) (*p =* 0.030) 3) VAS—EG: −21.18 (−29.83 to −12.53); CG: −11.84 (−20.11 to−3.57) (*p =* 0.084) 4) VPQ—EG: 4.04 (0.12 to 7.97); CG: 0.73 (−1.94 to 3.39) (*p =* 0.070) 5) SF-36 - SF-36 PCS—EG: 1.62 (−0.96 to 4.21); CG: 0.50 (−1.30 to 2.31) (0.717) - SF-36 MCS—EG: 0.53 (−2.69 to 3.75); CG: −0.26 (−2.92 to 2.40) (*p =* 0.680) 6) HADS - HADS-Anxiety—EG: −1.27 (−2.51 to −0.032); CG: −0.90 (−1.96 to 0.17) (*p =* 0.590) - HADS-Depression—EG: −0.68 (−1.57 to 0.21); CG: −0.21 (−1.11 to 0.69) (*p =* 0.486)	PSALTI significantly improved cough-related quality of life and objective cough frequency in comparison to the control group. No significant differences between groups were observed for changes in cough severity, voice outcomes and symptoms of anxiety and depression.
Sundar et al. (25)	Experimental: CPAP therapy Control: Sham CPAP therapy	**1) Cough- related QoL** 2) Sino-nasal Disease 3) Airway Inflammation Markers	**1) LCQ** 2) SNOT- 20 3) GERD-QoL 4) ALQ 5) Exhaled Breath Condensate	Pre/post scores 1) LCQ Total score—EG: 10.63 ± 3.94 vs. 17.24 ± 3.97; CG: 12.62 ± 4.13 vs. 14.69 ± 3.94 (*p =* 0.016) 2) SNOT-20—EG: 46 ± 14.8 vs. 29.77 ± 20.95; CG: 34.88 ± 14.63 vs. 26.44 ± 13.99 (*p =* 0.27) 3) GERD-QoL—EG: 9.44 ± 8.93 vs. 4.44 ± 4.85; CG: 6.33 ± 6.72 vs. 5.77 ± 7.66 (*p =* 0.27) 4) ALQ—EG: 8.88 ± 2.47 vs. 4.88 ± 2.47; CG: 7.44 ± 3.53 vs. 6.88 ± 3.05 (*p =* 0.09) 5) Exhaled Breath Condensate - NOX (umol/L)—EG: 3.34 ± 2.07 vs. 2.91 ± 2.32; CG: 3.35 ± 2.81 vs. 5.26 ± 0.18 (*p =* 0.258) - IL- 8 (pg/mL)—EG: 1.52 ± 1.41 vs. 1.00 ± 0.21; CG: 1.02 ± 0.24 vs. 1.04 ± 0.18 (*p =* 0.594) - 8iso (pg/mL)—EG: 4.92 ± 2.23 vs. 7.35 ± 3.47; CG: 3.99 ± 1.89 vs. 5.04 ± 2.13 (*p =* 0.156) - H_2_O_2_ nmol/L—EG: 2458.02 ± 324.88 vs. 1654.07 ± 239.71; CG: 1714.42 ± 337.1 vs. 1468.04 ± 143.58 (*p =* 0.643)	CPAP significantly improved cough-related quality of life in comparison to the control group. No significant differences between groups were observed for changes in the severity of sinonasal disease and airway inflammation markers
Kapela et al. (26)	Experimental: SPEICH-C + pre-recorded SLP technique videos Control: SPEICH-C	1) Cough- related QoL 2) Symptom and limitation outcomes 3) Voice outcomes **4) Accuracy performing the technique**	1) LCQ 2) Symptom severity and frequency rating scale 3) CAPE-V **4) Clinical Judgment (Correct vs. incorrect)**	Pre/Post Mean Differences 1) LCQ Total Score—EG: 15.3 ± 3.00 vs. 16.8 ± 2.70; CG: 11.20 ± 3.30 vs. 15.6 ± 2.40 (*p =* 0.796) 2) Symptom Frequency and Severity Total Score—EG: 25.9 ± 9.20 vs. 19.5 ± 9.20; CG: 22.5 ± 8.30 vs. 17.1 ± 7.50 (*p =* 0.941) 3) CAPE-V—EG: 21.9 ± 16.6 vs. 15.5 ± 12.5; CG: 9.80 ± 5.10 vs. 6.00 ± 3.30 (*p =* 0.575) 4) Rater judgement (Correct vs. Incorrect)—EG 7 vs. 1 vs. CG 7 vs. 0	No significant differences between groups were observed for changes in cough-related quality of life, symptom severity and frequency rating, voice outcomes, and technique performances.

*ACE, Angiotensin Converting Enzyme; ALQ, asthma life questionnaire; CAPE-V, Consensus of Auditory-Perceptual Evaluation of Voice; CG, Control Group; CPAP, Continuous Positive Airway Pressure; EBC, exhaled breath condensate measurements; EG, Experimental Group; GERD, Gastroesophageal Reflux Disease; GERD-QOL, GERD health-related quality of life questionnaire; HADS, Hospital Anxiety and Depression Scale; H_2_O_2_, Hydrogen Peroxide; IL-8, Interleukin-8; 8-isopg, 8-isoprostanes; LCM, Leicester Cough Monitor; LCQ, Leicester Cough Questionnaire; NOX, Nitrates/Nitrites; OSA; Obstructive Sleep Apnea; PSALTI, Physiotherapy, Speech and Language Therapy Intervention; PNDS, Postnasal Drip Syndrome; QoL, Quality of Life; SPEICH-C, Speech Pathology Evaluation and Intervention for Chronic Cough; SF-36, Short-form 36 Questionnaire; SNOT-20 sinonasal outcomes-20 questionnaire; Spielberg STAI, Spielberg State-Trait Anxiety Inventory; UACS, Upper Airway Cough Syndrome; VAS, Visual Analog Scale; VPQ, Vocal Performance Questionnaire*.

* Outcome measures presented in bolded format indicate the primary outcome measure of each article. Vertigan et al. (21) and Vertigan et al. (22) reported no primary outcome measure.

### Primary Outcome Measure—Cough-Related Quality of Life

**Figure 3:**
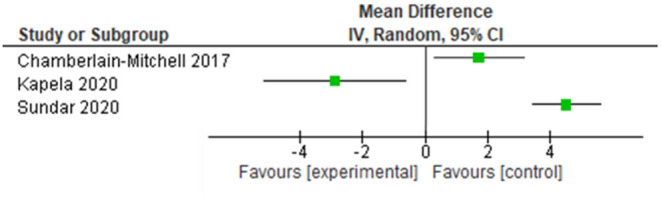
Effects of non-pharmacological interventions on cough-related quality of life. Date of publication of records included in the review.

Three articles provided data for cough-related quality of life immediately after the intervention using the LCQ (24–26). CPAP therapy (MD 4.54 95%CI 3.44 to 5.64), as well as the PSALTI (ES 1.53 95%CI 0.21 to 2.85) resulted in significant improvements on the LCQ total score compared with control groups (24, 25). Kapela et al. (26) showed that adding video recordings of breathing exercises to a standard intervention which included cough education, laryngeal irritation reduction and cough suppression strategies, and psycho-educational counseling, is not of added value (MD −2.90 95%CI −5.16 to −0.64) (26). One study reported on the mid-term effects (3 months) of PSALTI, showing no differences between the control and experimental group (MD 0.01 95%CI−1.62 to 1.64). Effects of these interventions on cough-related quality of life are in Figure 3.

### Objective Cough Measure

One study, objectively, evaluated cough counts using the Leicester Cough Monitor (LCM) (24), finding significant differences between the PSALTI and control groups for cough frequency (MD 0.59 95%CI 0.36 to 0.95 cough counts/hour) (24).

### Symptoms and Health-Related Quality of Life

Symptoms evaluated included breathing, voice, upper airway, and limitation symptoms with a Symptom Score (i.e., 5-point Likert scale) (21, 26), urge to cough with the Modified Borg Scale (21, 23), cough severity using the visual analog scale (24), and anxiety and depression using the Spielberger State–Trait Anxiety Inventory and the Hospital Anxiety and Depression scale (23). No difference in the Symptom Total Score was observed by the addition of a video of breathing exercises to a standard intervention including cough education, laryngeal irritation reduction and cough suppression strategies, and psycho-educational counseling (MD 1.00 95%CI −5.56 to 7.56 points) (26). Mindfulness (Median 0 IQR 0 to 2 points) and cough suppression (Median 0 IQR −1 to 1 points) were not superior to each other nor to the control group (Median 0 IQR −2 to 1 points) for improving participants' urge to cough (23). Symptoms of anxiety and depression did not change significantly with mindfulness or voluntary cough suppression (data not reported) (23), or PSALTI (HADS-Anxiety MD −0.42 95%CI −1.96 to 1.13 points; HADS-Depression MD −0.44 95%CI −1.69 to 0.81 points) (24).

Health-related quality of life was evaluated by the Short-Form 36 (SF-36) (24), the asthma life questionnaire (ALQ) (25) and the GERD health-related quality of life questionnaire (GERD-QOL) (25). The CPAP therapy and the PSALTI did not result in significant differences in health-related quality of life scores (ALQ MD −4.44 95%CI −7.18 to 1.70 points; GERD-QOL MD −3.44 95%CI −4.78 to 2.10 points; SF-36 physical component MD 0.56 95%CI −2.52 to 3.64 points; SF-36 mental component MD 0.81 95%CI −3.10 to 4.72 points) (24, 25).

### Voice

Three studies evaluated voice outcomes (21, 22, 24, 26) using the SLP's perceptual voice ratings, acoustic analysis, electroglottography (22), the vocal performance questionnaire (24), and the Consensus of Auditory-Perceptual Evaluation of Voice (CAPE-V) (26). The cough education, laryngeal irritation reduction and cough suppression strategies, and psycho-educational counseling multi-component therapy, delivered through speech-language therapy, resulted in significant improvements in the perceptual ratings of breathy, rough, strain, glottal fry voice (MD from 0.3 to 1 points) (22). No difference in the CAPE-V was observed between those attending the multi-component therapy delivered through speech-language therapy alone, or speech-language therapy paired with video recordings (26). No significant differences were observed between the PSALTI and control groups for changes in voice impairments (MD 3.9 95%CI −0.33 to 8.12 points) (24).

### Other Outcome Measures

Other outcome measures evaluated included the SLP's clinical judgement about the performance of the techniques (21) and effectiveness of the cough education, laryngeal irritation reduction, cough suppression strategies, psycho-educational counseling (rated as successful or partially successful or unsuccessful) (21), the accuracy of the patients' technique (26), the severity of sinonasal disease (25), and airway inflammatory markers from exhaled breath condensate. (25). The SLPs judged cough education, laryngeal irritation reduction, cough suppression strategies and psycho-educational counseling as significantly successful in improving outcomes compared to the control group (OR 48.13 95%CI 13.53 to 171.25) (21). Adding a video of breathing exercises to a standard SLP intervention resulted in no improvements to patient's accuracy in performing the SLP techniques. CPAP in the OSA population did not affect the sinonasal questionnaire scores (MD −7.79 95%CI −11.83 to −3.75 points) or airway inflammatory markers compared with sham-CPAP (25).

### Risk of Bias Assessment

**Figure 4:**
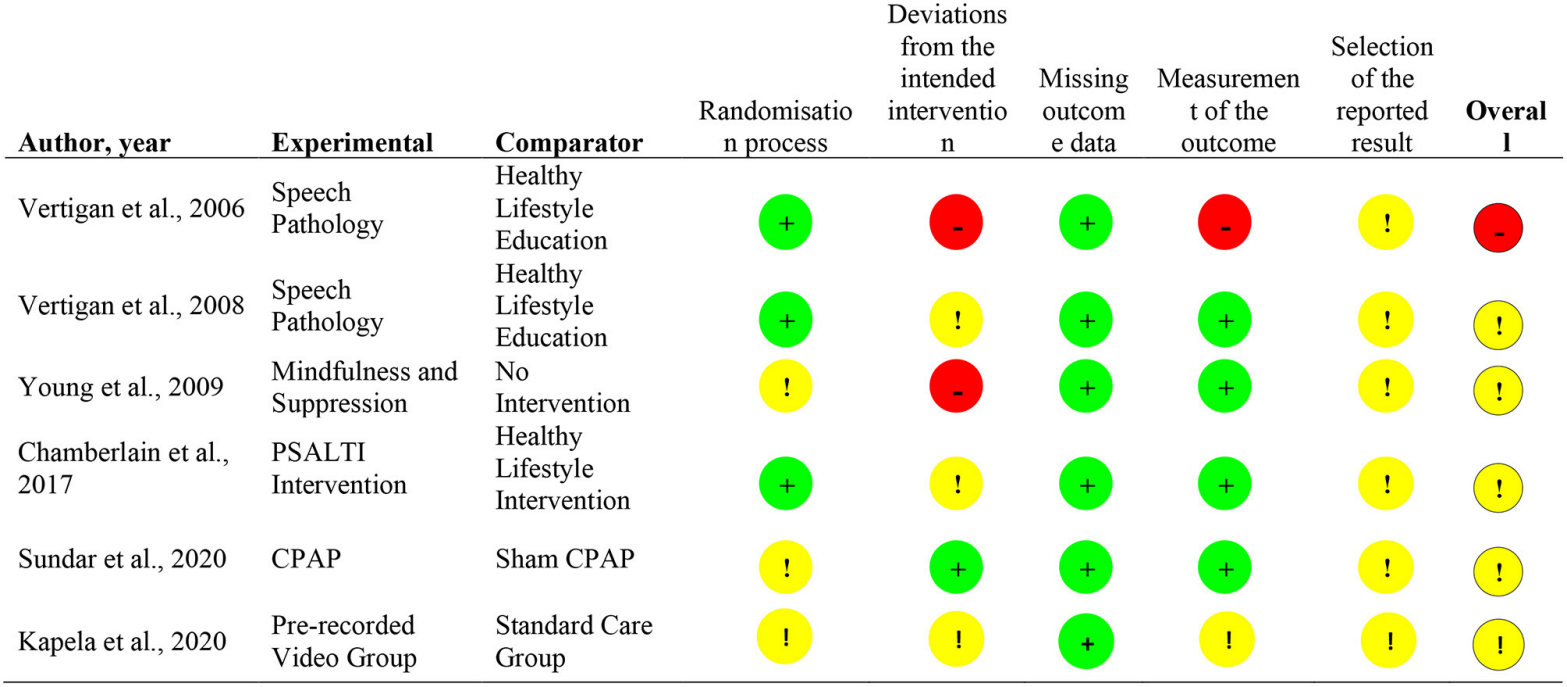
Risk of Bias Summary (1).

Most articles presented “some concerns” (*n* = 5) in the overall risk of bias (22–26), with one study presenting with a high overall risk of bias (21). The main source of bias emerged from the absence of studies' registration reporting on outcomes and planned analysis. Such absence prevented the establishment of conclusions about the selection or non/selection of reported outcomes and analyses. Two articles presented high risk of bias on the “deviations from intended interventions” domain (21, 23) and one study on the “measurement of the outcome” domain (3). Four of the included articles were single-blinded (21–24), one study was double-blinded (25), and one study did not blind the participants nor the investigators (26).The detailed risk of bias evaluation can be found in Figure 4.

## Discussion

Non-pharmacological cough therapies improved cough-specific health related quality of life (24, 25), cough frequency (24), and voice outcomes, such as breathy, rough, strain and glottal fry voice (22). No improvements were observed for urge to cough (24), cough severity (24, 26), anxiety and depression (23, 24), severity of sinonasal disease (25), or airway inflammatory markers (25). Small sample sizes, small effects, and large confidence intervals precluded confidence in establishing the impact of the identified nonpharmacological cough therapies. Variations in outcome measures and sampling times added to the study design heterogeneity, which prevented the pooling of results.

PTs and SLPs used similar therapies to treat non-productive chronic cough (i.e., education, laryngeal irritation reduction strategies, cough control, and psychoeducational strategies) (21, 22, 24, 26). The mechanism of actions of these multicomponent therapies is thought to be driven by a synergistic relationship between the various components to reduce sensory input triggering cough (27). For example, education provided basic knowledge of cough, which then increased the likelihood of cough control strategies being effective (27). Comparisons between single and multicomponent therapies could not be made, as time points varied, and no study specifically compared single vs. multicomponent therapies. CPAP did improve cough in those with OSA, possibly by its impact on lung inflation or on gastro-esophageal reflux (23, 26, 27), however, its use in other chronic respiratory diseases is unexplored. A recent systematic review explored multimodal treatments for refractory chronic cough and concluded that medical therapy, SLP, and procedural therapy all improve outcomes of chronic cough (9). In this review, all study designs were included and SLP was described as including a large number of interventions, such as physiotherapy and behavioral therapy and no distinction between them was performed, which could have influenced the conclusions presented (9). Our review compliments these results, by presenting the data from people with chronic respiratory diseases, including only the highest evidence available (RCTs) and differentiating between different disciplines and techniques performed for people with refractory chronic cough.

Of the 17 outcome measures that were used in the studies to evaluate cough interventions, 13 have adequately described their measurement properties for chronic cough and four have been validated for people with chronic respiratory diseases (19, 29–31). The LCQ and the LCM appear to be the most valid, reliable, and responsive measures (19), but they lack validation for cough associated with underlying chronic respiratory diseases (2, 6). The absence of disease-specific measures will also limit the extent to which the outcomes used may be applicable to underlying obstructive and interstitial lung disease (28, 31–33).

Despite three additional reviews published since 2010 looking at non-pharmacological management of chronic cough, this is the first systematic review in more than a decade to report on the effects of non-pharmacological cough therapies for, both, people with non-productive refractory cough and chronic respiratory diseases, and the results highlight the paucity of articles on this topic despite it being so prevalent. Nevertheless, this review is not without limitations. The quality of our findings was limited by the heterogeneity of the studies published. The duration of the interventions varied between 1 to 8 weeks. We acknowledge that a 1 week intervention may be unlikely to influence cough symptoms lasting for several years. Nevertheless, given the paucity of data in the field, and the uncertainty regarding the best design for providing non-pharmacological interventions, we decided to include all studies independently of frequency and duration of sessions to report on all the available evidence to date. Language competency limited our inclusion to studies in English, Portuguese and French. We excluded alternative medicine techniques, such as acupuncture and treatments that required ingestion of herbal medications, vitamins, and teas. We also excluded any therapies in which the use of pharmacological and non-pharmacologic treatments were paired. Lastly, comorbidities of articles reporting on individuals with refractory chronic cough included asthma, a chronic respiratory condition, except for Sundar et al. (25), in which chronic cough was not refractory, but attributed to the OSA. Results for patients with refractory chronic cough and asthma were not reported separately, and thus, no conclusions can be made regarding the effects of non-pharmacological therapy specifically for individuals with asthma. Furthermore, although therapies delivered through speech-language pathology and physiotherapy offer promising results as a form of nonpharmacological cough management, the long-term effects of this therapy are unknown and need to be further investigated.

### Implications for Research and Practice

Our findings highlight the need for relevant, well-designed studies in order to help guide clinicians to better manage refractory cough, both for individuals with no prior respiratory conditions, and those with documented underlying respiratory tract disorders. Prior to doing so, the optimal administration of non-pharmacological management strategies, as well as their role as part of dual pharmacologic and non-pharmacologic therapy, remains unclear.

## Conclusion

Non-pharmacological cough therapies improve cough-specific quality of life, cough frequency, and voice outcomes in some studies. Although their effectiveness alone or in combination with pharmacological therapies remains highly relevant, current evidence of effectiveness is insufficient for clinical recommendations to assist with the management of refractory cough or non-productive chronic cough associated with chronic respiratory diseases.

## Data Availability Statement

The original contributions presented in the study are included in the article/Supplementary Material, further inquiries can be directed to the corresponding author/s.

## Author Contributions

AI, AO, and DB contributed to conception and design of the study. AI, AO, RH, and YK conducted article screening. AI and AO organized the database, performed the statistical analysis, and wrote the first draft of the manuscript. DB, RG, and MK wrote sections of the manuscript through revisions. All authors contributed to manuscript revision, read, and approved the submitted version.

## Funding

DB holds a National Sanitarium Association (NSA) Chair in Respiratory/Pulmonary Rehabilitation Research. MK holds a Canada Research Chair in Critical Care Rehabiliation and Knowledge Translation.

## Conflict of Interest

The authors declare that the research was conducted in the absence of any commercial or financial relationships that could be construed as a potential conflict of interest.

## Publisher's Note

All claims expressed in this article are solely those of the authors and do not necessarily represent those of their affiliated organizations, or those of the publisher, the editors and the reviewers. Any product that may be evaluated in this article, or claim that may be made by its manufacturer, is not guaranteed or endorsed by the publisher.
