# Supplementary material for: Non-pharmacological Management of Non-productive Chronic Cough in Adults: A Systematic Review
Source: Front Rehabil Sci. 2022 May 26;3:905257. doi: 10.3389/fresc.2022.905257 (PMC9397766; doi:10.3389/fresc.2022.905257)
Supplement: Supplementary file 1 [file Table_1.DOCX]

Supplementary Material A: PRISMA 2020 Checklist

| **Section and Topic** | **Item #** | **Checklist item** | **Location where item is reported** |
| --- | --- | --- | --- |
| **TITLE** | | |  |
| Title | 1 | Identify the report as a systematic review. | Lines 1-2 |
| **ABSTRACT** | | |  |
| Abstract | 2 | See the PRISMA 2020 for Abstracts checklist. | Lines 41-67 |
| **INTRODUCTION** | | |  |
| Rationale | 3 | Describe the rationale for the review in the context of existing knowledge. | Lines 93-104 |
| Objectives | 4 | Provide an explicit statement of the objective(s) or question(s) the review addresses. | Lines 105-110 |
| **METHODS** | | |  |
| Eligibility criteria | 5 | Specify the inclusion and exclusion criteria for the review and how studies were grouped for the syntheses. | Lines 129-143 |
| Information sources | 6 | Specify all databases, registers, websites, organisations, reference lists and other sources searched or consulted to identify studies. Specify the date when each source was last searched or consulted. | Lines 120-127 |
| Search strategy | 7 | Present the full search strategies for all databases, registers and websites, including any filters and limits used. | Supplementary Material B |
| Selection process | 8 | Specify the methods used to decide whether a study met the inclusion criteria of the review, including how many reviewers screened each record and each report retrieved, whether they worked independently, and if applicable, details of automation tools used in the process. | Lines 145-153 |
| Data collection process | 9 | Specify the methods used to collect data from reports, including how many reviewers collected data from each report, whether they worked independently, any processes for obtaining or confirming data from study investigators, and if applicable, details of automation tools used in the process. | Lines 155-167 |
| Data items | 10a | List and define all outcomes for which data were sought. Specify whether all results that were compatible with each outcome domain in each study were sought (e.g. for all measures, time points, analyses), and if not, the methods used to decide which results to collect. | Lines 155-167 |
|  | 10b | List and define all other variables for which data were sought (e.g. participant and intervention characteristics, funding sources). Describe any assumptions made about any missing or unclear information. | Lines 155-167 |
| Study risk of bias assessment | 11 | Specify the methods used to assess risk of bias in the included studies, including details of the tool(s) used, how many reviewers assessed each study and whether they worked independently, and if applicable, details of automation tools used in the process. | Lines 169-175 |
| Effect measures | 12 | Specify for each outcome the effect measure(s) (e.g. risk ratio, mean difference) used in the synthesis or presentation of results. | Lines 179-183 |
| Synthesis methods | 13a | Describe the processes used to decide which studies were eligible for each synthesis (e.g. tabulating the study intervention characteristics and comparing against the planned groups for each synthesis (item #5)). | Table 2 |
|  | 13b | Describe any methods required to prepare the data for presentation or synthesis, such as handling of missing summary statistics, or data conversions. | Lines 177-183 |
|  | 13c | Describe any methods used to tabulate or visually display results of individual studies and syntheses. | Lines 156-158 |
|  | 13d | Describe any methods used to synthesize results and provide a rationale for the choice(s). If meta-analysis was performed, describe the model(s), method(s) to identify the presence and extent of statistical heterogeneity, and software package(s) used. | Lines 177-183 |
|  | 13e | Describe any methods used to explore possible causes of heterogeneity among study results (e.g. subgroup analysis, meta-regression). | Lines 177-183 |
|  | 13f | Describe any sensitivity analyses conducted to assess robustness of the synthesized results. | Lines 177-183 |
| Reporting bias assessment | 14 | Describe any methods used to assess risk of bias due to missing results in a synthesis (arising from reporting biases). | Lines 169-175 |
| Certainty assessment | 15 | Describe any methods used to assess certainty (or confidence) in the body of evidence for an outcome. | Line 179-183 |
| **RESULTS** | | |  |
| Study selection | 16a | Describe the results of the search and selection process, from the number of records identified in the search to the number of studies included in the review, ideally using a flow diagram. | Figure 1 |
|  | 16b | Cite studies that might appear to meet the inclusion criteria, but which were excluded, and explain why they were excluded. | Supplementary Material C |
| Study characteristics | 17 | Cite each included study and present its characteristics. | Table 1 |
| Risk of bias in studies | 18 | Present assessments of risk of bias for each included study. | Figure 4 |
| Results of individual studies | 19 | For all outcomes, present, for each study: (a) summary statistics for each group (where appropriate) and (b) an effect estimate and its precision (e.g. confidence/credible interval), ideally using structured tables or plots. | Table 3 |
| Results of syntheses | 20a | For each synthesis, briefly summarise the characteristics and risk of bias among contributing studies. | Lines 325-334 |
|  | 20b | Present results of all statistical syntheses conducted. If meta-analysis was done, present for each the summary estimate and its precision (e.g. confidence/credible interval) and measures of statistical heterogeneity. If comparing groups, describe the direction of the effect. | Lines 241-321 |
|  | 20c | Present results of all investigations of possible causes of heterogeneity among study results. | Lines 241-321 |
|  | 20d | Present results of all sensitivity analyses conducted to assess the robustness of the synthesized results. | Lines 241-321 |
| Reporting biases | 21 | Present assessments of risk of bias due to missing results (arising from reporting biases) for each synthesis assessed. | Lines 325-334 |
| Certainty of evidence | 22 | Present assessments of certainty (or confidence) in the body of evidence for each outcome assessed. | Figure 3 |
| **DISCUSSION** | | |  |
| Discussion | 23a | Provide a general interpretation of the results in the context of other evidence. | Lines 339-356 |
|  | 23b | Discuss any limitations of the evidence included in the review. | Lines 395-410 |
|  | 23c | Discuss any limitations of the review processes used. | Lines 377-381 |
|  | 23d | Discuss implications of the results for practice, policy, and future research. | Lines 381-389 |
| **OTHER INFORMATION** | | |  |
| Registration and protocol | 24a | Provide registration information for the review, including register name and registration number, or state that the review was not registered. | Lines 113-117 |
|  | 24b | Indicate where the review protocol can be accessed, or state that a protocol was not prepared. | Lines 113-117 |
|  | 24c | Describe and explain any amendments to information provided at registration or in the protocol. | / |
| Support | 25 | Describe sources of financial or non-financial support for the review, and the role of the funders or sponsors in the review. | Lines 25-27 |
| Competing interests | 26 | Declare any competing interests of review authors. | Lines 28 |
| Availability of data, code and other materials | 27 | Report which of the following are publicly available and where they can be found: template data collection forms; data extracted from included studies; data used for all analyses; analytic code; any other materials used in the review. | / |

*From:*  Page MJ, McKenzie JE, Bossuyt PM, Boutron I, Hoffmann TC, Mulrow CD, et al. The PRISMA 2020 statement: an updated guideline for reporting systematic reviews. BMJ 2021;372:n71. doi: 10.1136/bmj.n71

Supplementary Material B: Example of Search Strategy

MEDLINE Search Terms – to be performed in title, abstract and keywords

| **CONCEPT**: Chronic Respiratory Disease |
| --- |
| MeSh headings: exp respiratory tract diseases/ or exp bronchial diseases/ or exp asthma/ or bronchiectasis/ or bronchitis/ or exp bronchiolitis/ or exp bronchiolitis obliterans/ or exp tracheobronchomalacia/ or exp granuloma, respiratory tract/ or granuloma, laryngeal/ or laryngeal neoplasms/ or laryngomalacia/ or exp lung diseases/ or exp pulmonary aspergillosis/ or exp lung diseases, interstitial/ or exp alveolitis, extrinsic allergic/ or exp histiocytosis, langerhans-cell/ or exp lung diseases, obstructive/ or exp bronchitis/ or exp pulmonary disease, chronic obstructive/ or exp pneumoconiosis/ or exp silicosis/ or exp lung neoplasms/ or exp carcinoma, bronchogenic/ or pneumonia/ or exp bronchopneumonia/ or exp pulmonary fibrosis/ or exp idiopathic pulmonary fibrosis/ or exp idiopathic interstitial pneumonias/ or exp pleural diseases/ or exp pleural neoplasms/ or exp respiratory hypersensitivity/ or exp respiratory system abnormalities/ or exp respiratory tract neoplasms/ or exp bronchial neoplasms/ or exp thoracic diseases/ or exp tracheal diseases/ |
| **OR** |
| Key words: (COPD or (chronic adj3 bronchitis) or emphysema or ILD or respiratory diseas* or bronchial diseas* or asthm* or bronchiectas* or lung cancer or bronchitis or bronchiolitis or bronchiolitis obliterans or tracheobronchomalacia or granuloma, respiratory tract or laryngeal granuloma or laryngeal neoplasm* or laryngomalacia or lung disease* or pulmonary aspergillosis or alveolitis or Langerhans cell or (obstructive adj3 disease*) or chronic obstructive pulmonary disease or pneumoconiosis or silicosis or lung neoplasm* or carcinoma or pneumon* or bronchopneumon* or (idiopathic adj3 fibros*) or (idiopathic adj3 pneumon*) or pleural diseas* or pleural neoplasm* or respiratory hypersensitivity or (respiratory adj3 abnormalit*) or respiratory tract neoplasm* or bronchial neoplasm* or thoracic disease* or tracheal disease* or asbestosis or silicosis or beryliosis or pleuroparenchymal fibroelastosis or (interstitial adj3 disease) or granulomatous* or sclerosis or polymyositis or dermatomyositis or lupus or Hamman-Rich syndrome or bagassosis or histiocytosis).tw,kf. |

**AND**

| **CONCEPT**: Cough |
| --- |
| MeSh headings: Cough/ |
| **OR** |
| Key words: chronic cough*.tw,kf. |

**AND**

| **CONCEPT**: Non-pharmacological Interventions |
| --- |
| MeSh headings: exp Therapeutics/ or Airway Management/ or physical therapy modalities/ or exp exercise movement techniques/ or exp musculoskeletal manipulations/ or rehabilitation/ or exp exercise therapy/ or exp Speech Therapy/ or exp Complementary Therapies/ |
| **OR** |
| Key words: (therapeutic* or management or physiotherapy or speech thera* or language thera* or complementary thera* or alternative medicine or alternative thera* or intervention* or treatment* or rehabilitation* or nonpharmacologic*).tw,kf. |

Supplementary Material C: List of Excluded Articles

| **Reference** | **Reason for exclusion** |
| --- | --- |
| M. Zidan, H. Shaarawy, Assessment of the prevalence of obstructive sleep apnea in patients with undiagnosed chronic cough, Eur. Respir. J. 40 (2012). http://erj.ersjournals.com/content/40/Suppl_56/P1877.abstract?sid=22a4d8a0-8e17-4be5-b95d-3be3bf155dd9http://ovidsp.ovid.com/ovidweb.cgi?T=JS&PAGE=reference&D=emed13&NEWS=N&AN=71924234 NS  -. | Abstract in conference proceedings |
| A.B. Zakrisson, M. Arne, M. Hasselgren, K. Lisspers, B. Stallberg, K. Theander, A complex intervention of self-management for patients with COPD or CHF in primary care improved performance and satisfaction with regard to own selected activities; A longitudinal follow-up, J. Adv. Nurs. 75 (2019) 175‐186. https://doi.org/10.1111/jan.13899. | Abstract in conference proceedings |
| L. Yardley, J. Joseph, S. Michie, M. Weal, G. Wills, P. Little, Evaluation of a Web-based intervention providing tailored advice for self-management of minor respiratory symptoms: exploratory randomized controlled trial, J. Med. Internet Res. 12 (2010) e66. https://doi.org/10.2196/jmir.1599. | Abstract in conference proceedings |
| D. Xue, S. Han, S. Jiang, H. Sun, Y. Chen, Y. Li, W. Wang, Y. Feng, K. Wang, P. Li, Comprehensive geriatric assessment and traditional Chinese medicine intervention benefit symptom control in elderly patients with advanced non-small cell lung cancer, Med. Oncol. 32 (2015) 1–7. https://doi.org/10.1007/s12032-015-0563-5. | Abstract in conference proceedings |
| R.H. Wilson, S.M. Farber, W. Mandel, A new agent of therapeutic value in pulmonary insufficiency and irritative cough, Antibiot. Med. Clin. Ther. (New York, NY). 5 (1958) 567–572. http://ovidsp.ovid.com/ovidweb.cgi?T=JS&PAGE=reference&D=med1&NEWS=N&AN=13571961 NS  -. | Abstract in conference proceedings |
| S.H. Yu, A.M. Guo, X.J. Zhang, Effects of self-management education on quality of life of patients with chronic obstructive pulmonary disease, Int. J. Nurs. Sci. 1 (2014) 53‐57. https://doi.org/10.1016/j.ijnss.2014.02.014. | Case-series |
| J. Yorke, M. Lloyd-Williams, J. Smith, F. Blackhall, A. Harle, J. Warden, J. Ellis, M. Pilling, J. Haines, K. Luker, et al., Management of the respiratory distress symptom cluster in lung cancer: a randomised controlled feasibility trial, Support. Care Cancer. 23 (2015) 3373‐3384. https://doi.org/10.1007/s00520-015-2810-x. | Case-series |
| N. Yokohori, M. Hasegawa, A. Sato, H. Katsura, Severe sleep apnea syndrome associated with chronic cough without daytime sleepiness, Eur. Respir. J. 46 (2015). https://doi.org/http://dx.doi.org/10.1183/13993003.congress2015.PA3598. | Case-report |
| N. Yokohori, M. Hasegawa, A. Sato, H. Katsura, Utility of continuous positive airway pressure therapy for treating chronic coughs in patients with obstructive sleep apnea, Intern. Med. 53 (2014) 1079–1082. https://doi.org/http://dx.doi.org/10.2169/internalmedicine.53.1855. | Abstract in conference proceedings |
| P. Weiner, R. Magadle, M. Beckerman, M. Weiner, N. Berar-Yanay, Specific expiratory muscle training in COPD, Chest. 124 (2003) 468‐473. https://doi.org/10.1378/chest.124.2.468. | Observational Study |
| W. Wei, L. Yu, Y. Wang, X. Li, Z. Qiu, L. Wang, B. Liu, S. Liang, H. Lu, Z. Qiu, Efficacy and safety of modified sequential three-step empirical therapy for chronic cough, Respirology. 15 (2010) 830–836. https://doi.org/https://dx.doi.org/10.1111/j.1440-1843.2010.01785.x. | Pre-Post Design |
| D. Vilozni, M. Lavie, M. Ofek, I. Sarouk, O. Efrati, Cough Characteristics and FVC Maneuver in Cystic Fibrosis, Respir. Care. 59 (2014) 1912–1917. https://doi.org/10.4187/respcare.03290. | Case-report |
| S. Verver, M. Poelman, A. Bögels, S.L. Chisholm, F.W. Dekker, Effects of instruction by practice assistants on inhaler technique and respiratory symptoms of patients. A controlled randomized videotaped intervention study, Fam. Pract. 13 (1996) 35‐40. https://doi.org/10.1093/fampra/13.1.35. | Observational Study |
| A.E. Vertigan, D.G. Theodoros, P.G. Gibson, A.L. Winkworth, Voice and Upper Airway Symptoms in People With Chronic Cough and Paradoxical Vocal Fold Movement, J. Voice. 21 (2007) 361–383. https://doi.org/http://dx.doi.org/10.1016/j.jvoice.2005.12.008. | Case-report |
| A.E. Vertigan, S.M. Kapela, I. Franke, P.G. Gibson, The Effect of a Vocal Loading Test on Cough and Phonation in Patients With Chronic Cough, J. Voice. 31 (2017) 763–772. https://doi.org/https://dx.doi.org/10.1016/j.jvoice.2017.03.020. | Case-report |
| A.E. Vertigan, P.G. Gibson, Urge to cough and its application to the behavioural treatment of cough, Bratisl. Lek. Listy. 112 (2011) 102–108. http://ovidsp.ovid.com/ovidweb.cgi?T=JS&PAGE=reference&D=med8&NEWS=N&AN=21452759 NS  -. | Case-report |
| C.P. van der Schans, T.W. van der Mark, G. de Vries, D.A. Piers, H. Beekhuis, J.E. Dankert-Roelse, D.S. Postma, G.H. Koeter, Effect of positive expiratory pressure breathing in patients with cystic fibrosis, Thorax. 46 (1991) 252–256. http://ovidsp.ovid.com/ovidweb.cgi?T=JS&PAGE=reference&D=med3&NEWS=N&AN=2038733 NS  -. | Case-report |
| L. Wang, X. Kang, Z. Liu, L. Liu, S. Liu, Y. Bian, G. Li, Efficacy of acupuncture combined with Chinese herb in refractory chronic cough, Am. J. Respir. Crit. Care Med. 189 (2014). https://www.cochranelibrary.com/central/doi/10.1002/central/CN-01131442/full NS  -. | Case-report |
| R.D. Turner, G.H. Bothamley, Chronic cough and a normal chest X-ray-a simple systematic approach to exclude common causes before referral to secondary care: A retrospective cohort study, Npj Prim. Care Respir. Med. 26 (2016). https://doi.org/10.1038/npjpcrm.2015.81. | Retrospective Cohort |
| G. Taipin, C. Zukun, T. Xiantao, L. Zili, Z. Miansheng, T. Guo, Z. Chen, X. Tai, Z. Liu, M. Zhu, Space-time acupuncture for intractable cough after lupus nephropathy: A case report and literature review, Medicine (Baltimore). 96 (2017) 1–3. https://doi.org/10.1097/MD.0000000000009309. | Case-report |
| K.M. Sundar, S.E. Daly, A.M. Willis, A longitudinal study of CPAP therapy for patients with chronic cough and obstructive sleep apnoea, Cough. 9 (2013) 19. https://doi.org/http://dx.doi.org/10.1186/1745-9974-9-19. | Retrospective Cohort |
| K.M. Sundar, S.E. Daly, M.J. Pearce, W.T. Alward, Chronic cough and obstructive sleep apnea in a community-based pulmonary practice, Cough. 6 (2010). https://doi.org/10.1186/1745-9974-6-2. | Observational Study |
| K.M. Sundar, S.E. Daly, Clinical profile of chronic cough patients improving with therapy for sleep apnoea, Pulm. Pharmacol. Ther. 24 (2011) e4–e5. http://ovidsp.ovid.com/ovidweb.cgi?T=JS&PAGE=reference&D=emed12&NEWS=N&AN=70733466 NS  -. | Pharmacological intervention |
| K. Sundar, S. Daly, A. Willis, Prospective study of the prevalence of obstructive sleep apnea (OSA) in patients with chronic cough and impact of nocturnal continuous positive airway pressure therapy (CPAP) on the course of chronic cough, Chest. 142 (2012). https://doi.org/http://dx.doi.org/10.1378/chest.1387645. | Case-report |
| M. Sumitani, S. Nanjo, N. Miyamoto, S. Yoshida, M. Tsuda, M. Nishijima, S. Shoji, K. Tomii, N. Katakami, K. Miyamoto, Persistent dry cough effectively may be treated by soft extensible chest band, Chest. 142 (2012). https://doi.org/http://dx.doi.org/10.1378/chest.1388250. | Duration of cough not defined |
| N.C. Sullivan, Assessment and management of persistent cough in adults, Clin. Excell. Nurse Pract. 1 (1997) 417–422. http://libaccess.mcmaster.ca/login?url=http://search.ebscohost.com/login.aspx?direct=true&db=cin20&AN=107251100&site=ehost-live NS  -. | Duration of cough not defined |
| C.L. Su, L.L. Chiang, T.Y. Chiang, C.T. Yu, H.P. Kuo, H.C. Lin, Domiciliary positive expiratory pressure improves pulmonary function and exercise capacity in patients with chronic obstructive pulmonary disease, J. Formos. Med. Assoc. / Taiwan Yi Zhi. 106 (2007) 204‐211. https://doi.org/10.1016/S0929-6646(09)60241-2. | Duration of cough not defined |
| P. Sonnerfors, G. Faager, A.K. Nordlin, U. Einarsson, Pulmonary rehabilitation including interactive 3D visualization technique, in COPD, Eur. Respir. J. 54 (2019). https://doi.org/10.1183/13993003.congress-2019.PA569. | Pharmacological intervention |
| R.S. Soni, B. Ebersole, N. Jamal, Treatment of Chronic Cough, Otolaryngol. Head. Neck Surg. 156 (2017) 103–108. https://doi.org/https://dx.doi.org/10.1177/0194599816675299. | Duration of cough not defined |
| G. Simpson, Investigation and management of persistent dry cough, Thorax. 54 (1999) 469–470. http://ovidsp.ovid.com/ovidweb.cgi?T=JS&PAGE=reference&D=med4&NEWS=N&AN=10409073 NS  -. | Duration of cough not defined |
| S.W. Simon, Symptomatic treatment of asthmatic bronchitis, J. Am. Geriatr. Soc. 8 (1960) 107–111. http://ovidsp.ovid.com/ovidweb.cgi?T=JS&PAGE=reference&D=med1&NEWS=N&AN=14446944 NS  -. | Pharmacological intervention |
| M. Tomruk, E. Keles, S. Ozalevli, A.O. Alpaydin, Effects of thoracic kinesio taping on pulmonary functions, respiratory muscle strength and functional capacity in COPD patients: a pilot randomized controlled study, Eur. Respir. J. 50 (2017). https://doi.org/10.1183/1393003.congress-2017.PA1534. | Secondary source |
| T. Toljamo, M. Kaukonen, P. Nieminen, V.L. Kinnula, Early detection of COPD combined with individualized counselling for smoking cessation: a two-year prospective study, Scand. J. Prim. Health Care. 28 (2010) 41–46. https://doi.org/10.3109/02813431003630105. | Duration of cough not defined |
| S.J. Stenekes, A. Hughes, M.-C. Gregoire, G. Frager, W.M. Robinson, P.J. McGrath, Frequency and self-management of pain, dyspnea, and cough in cystic fibrosis, J. Pain Symptom Manage. 38 (2009) 837–848. https://doi.org/https://dx.doi.org/10.1016/j.jpainsymman.2009.04.029. | No chronic cough (defined as >=8weeks) |
| A.C. Shembel, C.A. Rosen, T.G. Zullo, J.L. Gartner-Schmidt, Development and validation of the cough severity index: A severity index for chronic cough related to the upper airway, Laryngoscope. 123 (2013) 1931–1936. https://doi.org/http://dx.doi.org/10.1002/lary.23916. | Duration of cough not defined |
| J.C. Schraa, J.F. Dirks, Hypnotic treatment of the alexithymic patient: a case report, Am. J. Clin. Hypn. 23 (1981) 207–210. http://ovidsp.ovid.com/ovidweb.cgi?T=JS&PAGE=reference&D=med2&NEWS=N&AN=7246472 NS  -. | Duration of cough not defined |
| L. Slovarp, B.K. Loomis, A. Glaspey, Assessing referral and practice patterns of patients with chronic cough referred for behavioral cough suppression therapy, Chronic Respir. Dis. 15 (2018) 296–305. https://doi.org/10.1177/1479972318755722. | Duration of cough not defined |
| P. Sivasothy, L. Brown, I.E. Smith, J.M. Shneerson, Effect of manually assisted cough and mechanical insufflation on cough flow of normal subjects, patients with chronic obstructive pulmonary disease (COPD), and patients with respiratory muscle weakness, Thorax. 56 (2001) 438–444. http://ovidsp.ovid.com/ovidweb.cgi?T=JS&PAGE=reference&D=med4&NEWS=N&AN=11359958 NS  -. | Duration of cough not defined |
| B. Sitkauskiene, K. Stravinskaite, R. Sakalauskas, P. V Dicpinigaitis, Changes in cough reflex sensitivity after cessation and resumption of cigarette smoking, Pulm. Pharmacol. Ther. 20 (2007) 240–243. http://ovidsp.ovid.com/ovidweb.cgi?T=JS&PAGE=reference&D=med6&NEWS=N&AN=17045500 NS  -. | Duration of cough not defined |
| V. Singh, D.C. Khandelwal, R. Khandelwal, S. Abusaria, Pulmonary rehabilitation in patients with chronic obstructive pulmonary disease, Indian J. Chest Dis. Allied Sci. 45 (2003) 13‐17. https://www.cochranelibrary.com/central/doi/10.1002/central/CN-00430952/full NS  -. | Paediatric population |
| J. Selby, E. Bailey, F. Gillies, J.H. Hull, Time to re-group: a novel approach to the delivery of speech and language therapy for chronic refractory cough, Thorax. 72 (2017) A141‐. https://doi.org/10.1136/thoraxjnl-2017-210983.249. | No chronic cough (defined as >=8weeks) |
| G. Rutten, J. Van Eijk, M. Beek, H. Van der Velden, Patient education about cough: effect on the consulting behaviour of general practice patients, Br. J. Gen. Pract. 41 (1991) 289‐292. https://www.cochranelibrary.com/central/doi/10.1002/central/CN-00080116/full NS  -. | Intervention not reported |
| L. Rose, D. McKim, D. Leasa, M. Nonoyama, A. Tandon, M. Kaminska, C. O’Connell, A. Loewen, B. Connolly, P. Murphy, N. Hart, J. Road, Monitoring Cough Effectiveness and Use of Airway Clearance Strategies: A Canadian and UK Survey, Respir. Care. 63 (2018) 1506–1513. https://doi.org/https://dx.doi.org/10.4187/respcare.06321. | Duration of cough not defined |
| B. Riegel, J.E. Warmoth, S.J. Middaugh, W.G. Kee, L.C. Nicholson, D.M. Melton, D.K. Parikh, J.C. Rosenberg, Psychogenic cough treated with biofeedback and psychotherapy. A review and case report, Am. J. Phys. Med. Rehabil. 74 (1995) 155–158. http://ovidsp.ovid.com/ovidweb.cgi?T=JS&PAGE=reference&D=med3&NEWS=N&AN=7710731 NS  -. | Duration of cough not defined |
| M. Ribeiro, C.A. De Castro Pereira, L.E. Nery, O.S. Beppu, C.O. Silva, A prospective longitudinal study of clinical characteristics, laboratory findings, diagnostic spectrum and outcomes of specific therapy in adult patients with chronic cough in a general respiratory clinic, Int. J. Clin. Pract. 60 (2006) 799–805. https://doi.org/http://dx.doi.org/10.1111/j.1368-5031.2006.00876.x. | Duration of cough not defined |
| A.A. Raj, D.I. Pavord, S.S. Birring, Clinical cough IV:what is the minimal important difference for the Leicester Cough Questionnaire?, Handb. Exp. Pharmacol. (2009) 311–320. https://doi.org/https://dx.doi.org/10.1007/978-3-540-79842-2_16. | Duration of cough not defined |
| R.H. Poe, R. V Harder, R.H. Israel, M.C. Kallay, Chronic persistent cough. Experience in diagnosis and outcome using an anatomic diagnostic protocol, Chest. 95 (1989) 723–728. http://ovidsp.ovid.com/ovidweb.cgi?T=JS&PAGE=reference&D=med3&NEWS=N&AN=2924600 NS  -. | Pharmacological intervention |
| C. Pisinger, N.S. Godtfredsen, T. Jørgensen, Smoking reduction and cessation reduce chronic cough in a general population: the Inter99 study, Clin Respir J. 2 (2008) 41–46. https://doi.org/10.1111/j.1752-699x.2007.00029.x. | No chronic cough (defined as >=8weeks) |
| A.S. Patel, G. Watkin, B. Willig, K. Mutalithas, H. Bellas, R. Garrod, I.D. Pavord, S.S. Birring, Improvement in health status following cough-suppression physiotherapy for patients with chronic cough, Chron. Respir. Dis. 8 (2011) 253–258. https://doi.org/https://dx.doi.org/10.1177/1479972311422547. | Cough augmentation |
| A.D. Palmer, R.K. Bolognone, S. Thomsen, D. Britton, J. Schindler, D.J. Graville, The Safety and Efficacy of Expiratory Muscle Strength Training for Rehabilitation After Supracricoid Partial Laryngectomy: A Pilot Investigation, Ann. Otol. Rhinol. Laryngol. 128 (2019) 169–176. https://doi.org/https://dx.doi.org/10.1177/0003489418812901. | Duration of cough not defined |
| H.J. Pai, R.S. Azevedo, A.L.F. Braga, L.C. Martins, B.M. Saraiva-Romanholo, M. de A. Martins, C.A. Lin, A randomized, controlled, crossover study in patients with mild and moderate asthma undergoing treatment with traditional Chinese acupuncture, Clinics (Sao Paulo). 70 (2015) 663–669. https://doi.org/https://dx.doi.org/10.6061/clinics/2015(10)01. | No chronic cough (defined as >=8weeks) |
| Nct, Sham CPAP vs. Straight CPAP for Chronic Cough, Https://Clinicaltrials.Gov/Show/NCT03172130. (2017). https://www.cochranelibrary.com/central/doi/10.1002/central/CN-01594649/full NS  -. | Duration of cough not defined |
| Nct, Cystic Fibrosis- Children and Adults Tai Chi Study, Https://Clinicaltrials.Gov/Show/NCT02054377. (2014). https://www.cochranelibrary.com/central/doi/10.1002/central/CN-01543468/full NS  -. | Pharmacological intervention |
| Nct, Does CBT Improve the Perception/Impact of Cough and Breathlessness in IPF Patients, Https://Clinicaltrials.Gov/Show/NCT01738711. (2012). https://www.cochranelibrary.com/central/doi/10.1002/central/CN-01539028/full NS  -. | Duration of cough not defined |
| P.R. Munford, D. Reardon, R.P. Liberman, L. Allen, Behavioral treatment of hysterical coughing and mutism: a case study, J. Consult. Clin. Psychol. 44 (1976) 1008–1014. http://ovidsp.ovid.com/ovidweb.cgi?T=JS&PAGE=reference&D=med1&NEWS=N&AN=11229 NS  -. | No intervention |
| Nct, Cough Desensitization Therapy for Cough Hypersensitivity Syndrome, Https://Clinicaltrials.Gov/Show/NCT04256733. (2020). https://www.cochranelibrary.com/central/doi/10.1002/central/CN-02080113/full NS  -. | No chronic cough (defined as >=8weeks) |
| Nct, Feasibility & Effect of a Tele-rehabilitation Program in Pulmonary Sarcoidosis Pulmonary Sarcoidosis, Https://Clinicaltrials.Gov/Show/NCT03914027. (2019). https://www.cochranelibrary.com/central/doi/10.1002/central/CN-01931282/full NS  -. | Duration of cough not defined |
| A.H. Morice, S. Faruqi, C.E. Wright, R. Thompson, J.M. Bland, Cough hypersensitivity syndrome: a distinct clinical entity, Lung. 189 (2011) 73–79. https://doi.org/https://dx.doi.org/10.1007/s00408-010-9272-1. | Pharmacological intervention |
| F.J. Molina-Saldarriaga, N.J. Fonseca-Ruiz, D.P. Cuesta-Castro, A. Esteban, F. Frutos-Vivar, Spontaneous breathing trial in chronic obstructive pulmonary disease: continuous positive airway pressure (CPAP) versus T-piece, Med. Intensiva. 34 (2010) 453‐458. https://doi.org/10.1016/j.medin.2010.03.007. | Pharmacological intervention |
| S.J. Mohammed, J. Steer, J. Ellis, L. Kellett, N. Kurji-Smith, S.M. Parker, Non-pharmacological cough suppression therapy for cough associated with underlying lung disease, Thorax. 73 (2018) A98–A99. https://doi.org/http://dx.doi.org/10.1136/thorax-2018-212555.164. | Trial registration |
| S. Mohammed, J. Steer, J. Ellis, S.M. Parker, Nonpharmacological cough control therapy for chronic refractory cough and cough associated with underlying lung disease, ERJ Open Res. 6 (2020) 243–2019. https://doi.org/http://dx.doi.org/10.1183/23120541.00243-2019. | Duration of cough not defined |
| A. Miles, M. Jardine, F. Johnston, M. de Lisle, P. Friary, J. Allen, Effect of Lee Silverman Voice Treatment (LSVT LOUD R) on swallowing and cough in Parkinson’s disease: A pilot study, J. Neurol. Sci. 383 (2017) 180–187. https://doi.org/https://dx.doi.org/10.1016/j.jns.2017.11.015. | Cough augmentation |
| H.C. Long, M.H. Zhang, L. Yu, X.H. Li, X.F. Zhou, B.R. Xiao, Y.J. Liu, Z.L. Wang, C.T. Liu, A pilot study of a simple management strategy for patients with chronic cough in Chengdu, Respirology. 17 (2012) 12. https://doi.org/http://dx.doi.org/10.1111/j.1440-1843.2012.02288.x. | Intervention not reported |
| S.F. Lillie, J. Haines, A. Vyas, S.J. Fowler, Speech and language therapy by SkypeTM for vocal cord dysfunction and chronic cough, Thorax. 69 (2014) A126–A127. https://doi.org/http://dx.doi.org/10.1136/thoraxjnl-2014-206260.253. | Duration of cough not defined |
| D. Masmoudi, A. Krid, S. Chakroun, H. Trabelsi, L. Triki, H. Zouari, I. Kammoun, K. Masmoudi, M. Chaabouni, Does continuous positive airway pressure improve the sub-maximal exercise capacity in obese patients with severe obstructive sleep-apnea-hypopnea-syndrome ?, Eur. Respir. J. 54 (2019). https://doi.org/http://dx.doi.org/10.1183/13993003.congress-2019.PA4173. | Trial registration |
| D.J. Maselli, J.I. Peters, In refractory chronic cough, physiotherapy plus speech and language therapy improved quality of life at 4 weeks, Ann. Intern. Med. 166 (2017) JC3. https://doi.org/http://dx.doi.org/10.7326/ACPJC-2017-166-2-003. | Duration of cough not defined |
| D. Marimuthu, F. Nasir, B. Singh, Laryngeal Amyloidosis: A Case Report, Int. Med. J. 26 (2019) 51–52. http://libaccess.mcmaster.ca/login?url=http://search.ebscohost.com/login.aspx?direct=true&db=cin20&AN=134551668&site=ehost-live NS  -. | Duration of cough not defined |
| B.M. Levine, Systematic evaluation and treatment of chronic cough in a community setting, Allergy Asthma Proc. 29 (2008) 336–342. https://doi.org/http://dx.doi.org/10.2500/aap.2008.29.3122. | Cough augmentation |
| S.T. Kulnik, S.S. Birring, J. Moxham, G.F. Rafferty, L. Kalra, Does respiratory muscle training improve cough flow in acute stroke? Pilot randomized controlled trial, Stroke (00392499). 46 (2015) 447–453. https://doi.org/10.1161/STROKEAHA.114.007110. | No chronic cough (defined as >=8weeks) |
| K. Krakowiak, M. Dabrowska, F. Bula, A. Lobacz, D. Rojek, E.M. Grabczak, A. Krzeski, R. Krenke, Speech therapy - a non-pharmacological method to manage difficult-to-treat chronic cough, Adv. Respir. Med. 85 (2017) 116–120. https://doi.org/http://dx.doi.org/10.5603/ARM.2017.0018. | Duration of cough not defined |
| J. Krahnke, D. Gentile, B. Angelini, M. Danzig, D. Skoner, Comparison of objective and subjective measurements of cough frequency in patients with seasonal allergic rhinitis, Ann. Allergy, Asthma Immunol. 93 (2004) 259‐264. https://doi.org/10.1016/S1081-1206(10)61498-6. | Duration of cough not defined |
| J. Lee, M. Kim, J.H. Kim, Y.R. Lee, S. Kim, Y. Kim, A cheaper, faster way to resolve chronic cough, J. Fam. Pract. 56 (2007) 641–646. http://ovidsp.ovid.com/ovidweb.cgi?T=JS&PAGE=reference&D=med6&NEWS=N&AN=17669289 NS  -. | Pharmacological intervention |
| A.L. Lee, C.J. Hill, N. Cecins, S. Jenkins, C.F. McDonald, A.T. Burge, L. Rautela, R.G. Stirling, P.J. Thompson, A.E. Holland, The short and long term effects of exercise training in non-cystic fibrosis bronchiectasis--a randomised controlled trial, Respir. Res. 15 (2014) 44. https://doi.org/10.1186/1465-9921-15-44. | Pharmacological intervention |
| A. Lee, C. Hill, N. Cecins, S. Jenkins, C. McDonald, A. Burge, L. Rautela, R. Stirling, P. Thompson, A. Holland, Exercise training is beneficial in patients with noncystic fibrosis bronchiectasis A multicentre, randomised controlled trial, Eur. Respir. J. 40 (2012). https://www.cochranelibrary.com/central/doi/10.1002/central/CN-01084722/full NS  -. | No chronic cough (defined as >=8weeks) |
| H.N. Le, H.T. Chu, Evaluating the effectiveness of therapeutic intervention counseling in patients with chronic obstructive pulmonary disease in nghe an province, Respirology. 24 (2019) 156‐. https://www.cochranelibrary.com/central/doi/10.1002/central/CN-02119765/full NS  -. | Duration of cough not defined |
| F. Lavorini, G.A. Fontana, E. Chellini, C. Magni, R. Duranti, J. Widdicombe, Desensitization of the cough reflex by exercise and voluntary isocapnic hyperpnea, J. Appl. Physiol. 108 (2010) 1061–1068. https://doi.org/https://dx.doi.org/10.1152/japplphysiol.00423.2009. | Duration of cough not defined |
| Z. Lanfang, W. Yanni, D. Mengya, H. Lian, X. Guozhu, W. Hongmei, Z. Chunlan, C. Peijuan, Assessment and management of cough among patients with lung cancer in a radiotherapy department in China: a best practice implementation project, JBI Database Syst. Rev. Implement. Reports. 17 (2019) 2390–2400. https://doi.org/10.11124/JBISRIR-2017-004001. | Wrong language |
| J. Kim, P. Davenport, C. Sapienza, Effect of expiratory muscle strength training on elderly cough function, Arch. Gerontol. Geriatr. 48 (2009) 361–366. https://doi.org/https://dx.doi.org/10.1016/j.archger.2008.03.006. | Duration of cough not defined |
| S. Khoshkesht, M. Zakerimoghadam, S. Ghiyasvandian, A. Kazemnejad, M. Hashemian, The effect of home-based pulmonary rehabilitation on self-efficacy in chronic obstructive pulmonary disease patients, J. Pak. Med. Assoc. 65 (2015) 1041‐1046. https://www.cochranelibrary.com/central/doi/10.1002/central/CN-01264691/full NS  -. | Cough augmentation |
| P.C. Jin, X.L. Fang, Wentong needling method in the treatment of 30 cases with long-term cough after common cold, World J. Acupunct. - Moxibustion. 23 (2013) 62–65. https://doi.org/http://dx.doi.org/10.1016/S1003-5257%2813%2960064-1. | Duration of cough not defined |
| A. Ing, Chronic cough, Respirology. 2 (1997) 309‐316. https://doi.org/10.1111/j.1440-1843.1997.tb00095.x. | Secondary source |
| J. Hu, Acupuncture treatment of cough, J. Tradit. Chinese Med. = Chung i Tsa Chih Ying Wen Pan. 27 (2007) 233–235. http://ovidsp.ovid.com/ovidweb.cgi?T=JS&PAGE=reference&D=med6&NEWS=N&AN=17955664 NS  -. | Duration of cough not defined |
| S.S. Jacobs, J.J. Swigris, G. Rosen, Interstitial lung disease patient-reported cough management strategies: Don’t move, don’t talk, Am. J. Respir. Crit. Care Med. 189 (2014). http://www.atsjournals.org/doi/pdf/10.1164/ajrccm-conference.2014.189.1_MeetingAbstracts.A2455http://ovidsp.ovid.com/ovidweb.cgi?T=JS&PAGE=reference&D=emed15&NEWS=N&AN=72041984 NS  -. | Duration of cough not defined |
| M. Izumi, S. Akifusa, S. Ganaha, Y. Yamashita, Activities of daily living decline is a predictor of lowered coughing ability and correlates with rehabilitative effect of tongue cleaning on coughing ability, Odontology. 107 (2019) 393‐400. https://doi.org/10.1007/s10266-019-00415-9. | Pharmacological intervention |
| R.S. Irwin, M.R. Pratter, P.S. Holland, R.W. Corwin, J.P. Hughes, Postnasal drip causes cough and is associated with reversible upper airway obstruction, Chest. 85 (1984) 346–352. http://ovidsp.ovid.com/ovidweb.cgi?T=JS&PAGE=reference&D=med2&NEWS=N&AN=6697790 NS  -. | Pharmacological intervention |
| S.A.M. Heikkinen, E.M.S. Makikyro, T.T. Hugg, M.S. Jaakkola, J.J.K. Jaakkola, Effects of regular exercise on asthma control in young adults, J. Asthma. 55 (2018) 726–733. https://doi.org/https://dx.doi.org/10.1080/02770903.2017.1366510. | No intervention |
| V. Hatzelis, T. Murry, Paradoxical vocal fold motion: respiratory retraining to manage long-term symptoms, J. Soc. Bras. Fonoaudiol. 24 (2012) 80–85. http://ovidsp.ovid.com/ovidweb.cgi?T=JS&PAGE=reference&D=emed13&NEWS=N&AN=365836541 NS  -. | Duration of cough not defined |
| A. Hasani, D. Pavia, J.E. Agnew, S.W. Clarke, Regional mucus transport following unproductive cough and forced expiration technique in patients with airways obstruction, Chest. 105 (1994) 1420–1425. http://libaccess.mcmaster.ca/login?url=http://search.ebscohost.com/login.aspx?direct=true&db=cin20&AN=136613811&site=ehost-live NS  -. | Pharmacological intervention |
| A. Gross, P. Hortig, S. Darb-Esfahani, T. Schneider, Chronic cough, pleuritic chest pain, and night sweats in a 45‑year-old female smoker, Internist. 57 (2016) 1126–1131. https://doi.org/10.1007/s00108-016-0105-z. | Duration of cough not defined |
| S.H. Jang, J.H. Kim, H.K. Koo, I. Jeong, S.Y. Park, D.G. Kim, C.K. Rhee, Y.H. Kim, S.K. Kim, E.Y. Choi, J.Y. Moon, J.W. Shin, J.W. Kim, K.H. Min, D.K. Kim, H. Lee, H.K. Yoon, H.J. Kim, Development and first validation of the cough assessment test (COAT), Eur. Respir. J. 50 (2017). https://doi.org/http://dx.doi.org/10.1183/1393003.congress-2017.PA3894. | Secondary source |
| K. Hall, S. Kuys, P. Masel, K. Fong, I. Yang, Outpatient physiotherapy improves quality of life for non CF bronchiectasis, Respirology. 19 (2014) 96. https://doi.org/http://dx.doi.org/10.1111/resp.12263. | Duration of cough not defined |
| J. Haines, A. Vyas, C. Slinger, S. Lillie, S.J. Fowler, Speech and language therapy for chronic cough and vocal cord dysfunction: Patient satisfaction with therapy given facetoface and via videocalls, Eur. Respir. J. 46 (2015). https://doi.org/http://dx.doi.org/10.1183/13993003.congress2015.PA3944. | Duration of cough not defined |
| J. Haines, A. Vyas, C. Slinger, N. Cheyne, S.J. Fowler, Clinical characteristics and management of patients presenting to the “airways clinic”; a specialised tertiary multi-disciplinary respiratory service, Thorax. 70 (2015) A232. https://doi.org/http://dx.doi.org/10.1136/thoraxjnl-2015-207770.440. | Pharmacological intervention |
| T. Furness, N. Bate, L. Welsh, G. Naughton, C. Lorenzen, Efficacy of WBV to improve functional performance of people with COPD, J. Sci. Med. Sport. 15 (2012) S361. https://doi.org/http://dx.doi.org/10.1016/j.jsams.2012.11.879. | Duration of cough not defined |
| T. Furness, N. Bate, L. Welsh, G. Naughton, C. Lorenzen, Efficacy of a whole-body vibration intervention to effect exercise tolerance and functional performance of the lower limbs of people with chronic obstructive pulmonary disease, BMC Pulm. Med. 12 (2012). https://doi.org/10.1186/1471-2466-12-71. | Pharmacological intervention |
| T. Furness, N. Bate, B. Browne, G. Naughton, C. Lorenzen, Safety of a single WBV session for people with COPD, J. Sci. Med. Sport. 15 (2012) S149. https://doi.org/http://dx.doi.org/10.1016/j.jsams.2012.11.361. | Duration of cough not defined |
| S.K. Field, D.P. Conley, A.M. Thawer, R. Leigh, R.L. Cowie, Effect of the management of patients with chronic cough by pulmonologists and certified respiratory educators on quality of life: a randomized trial, Chest. 136 (2009) 1021‐1028. https://doi.org/10.1378/chest.08-2399. | Pharmacological intervention |
| S.K. Field, D.P. Conley, A.M. Thawer, R. Leigh, R.L. Cowie, Assessment and management of patients with chronic cough by Certified Respiratory Educators: a randomized controlled trial, Can. Respir. J. 16 (2009) 49‐54. https://doi.org/10.1155/2009/263054. | Trial registration |
| S.K. Field, D.P. Conley, A.M. Thawer, R. Leigh, R.L. Cowie, Effects at 6 months of the management of chronic cough patients by pulmonologists and certified asthma educators on quality of life: a randomized trial, Chest. 134 (2008) 17002s. https://www.cochranelibrary.com/central/doi/10.1002/central/CN-00718287/full NS  -. | Pharmacological intervention |
| S.K. Field, D. Conley, A. Thawer, R. Leigh, R.L. Cowie, Management of chronic cough patients by certified asthma educators CAE): a randomized trial, Am. Thorac. Soc. Int. Conf. May 16-21, 2008, Toronto. (2008) Poster #E130. https://www.cochranelibrary.com/central/doi/10.1002/central/CN-00677037/full NS  -. | Secondary source |
| S. Faruqi, A. Fahim, A.H. Morice, Chronic cough and obstructive sleep apnoea: reflux-associated cough hypersensitivity?, Eur. Respir. J. 40 (2012) 1049–1050. http://ovidsp.ovid.com/ovidweb.cgi?T=JS&PAGE=reference&D=med9&NEWS=N&AN=23024327 NS  -. | Duration of cough not defined |
| C.T. French, R.S. Irwin, K.E. Fletcher, T.M. Adams, Evaluation of a cough-specific quality-of-life questionnaire, Chest. 121 (2002) 1123–1131. http://ovidsp.ovid.com/ovidweb.cgi?T=JS&PAGE=reference&D=med4&NEWS=N&AN=11948042 NS  -. | Pharmacological intervention |
| C.L. French, R.S. Irwin, F.J. Curley, C.J. Krikorian, Impact of chronic cough on quality of life, Arch. Intern. Med. 158 (1998) 1657–1661. http://ovidsp.ovid.com/ovidweb.cgi?T=JS&PAGE=reference&D=med4&NEWS=N&AN=9701100 NS  -. | Intervention not reported |
| M.L. Franchini, R. Athanazio, L.F. Amato-Lourenco, W. Carreirao-Neto, P.H.N. Saldiva, G. Lorenzi-Filho, B.K. Rubin, N.K. Nakagawa, Oxygen With Cold Bubble Humidification Is No Better Than Dry Oxygen in Preventing Mucus Dehydration, Decreased Mucociliary Clearance, and Decline in Pulmonary Function, Chest. 150 (2016) 407–414. https://doi.org/https://dx.doi.org/10.1016/j.chest.2016.03.035. | No chronic cough (defined as >=8weeks) |
| S.K. Epstein, Noninvasive ventilation to shorten the duration of mechanical ventilation, Respir. Care. 54 (2009) 198‐208. https://www.cochranelibrary.com/central/doi/10.1002/central/CN-01745418/full NS  -. | Duration of cough not defined |
| C. Emirgil, B.J. Sobol, J. Norman, E. Moskowitz, P. Goyal, B. Wadhwani, A study of the long-term effect of therapy in chronic obstructive pulmonary disease, Am. J. Med. 47 (1969) 367–377. http://ovidsp.ovid.com/ovidweb.cgi?T=JS&PAGE=reference&D=med1&NEWS=N&AN=4897276 NS  -. | Pharmacological intervention |
| J. Ellis, R. Wagland, C. Tishelman, M.L. Williams, C.D. Bailey, J. Haines, A. Caress, P. Lorigan, J.A. Smith, R. Booton, F. Blackhall, A. Molassiotis, Considerations in developing and delivering a nonpharmacological intervention for symptom management in lung cancer: the views of patients and informal caregivers, J. Pain Symptom Manage. 44 (2012) 831–842. https://doi.org/https://dx.doi.org/10.1016/j.jpainsymman.2011.12.274. | Duration of cough not defined |
| E.Ö. Efraimsson, C. Hillervik, A. Ehrenberg, Effects of COPD self-care management education at a nurse-led primary health care clinic, Scand. J. Caring Sci. 22 (2008) 178–185. https://doi.org/10.1111/j.1471-6712.2007.00510.x. | Trial registration |
| T.M.L. Eagan, A. Gulsvik, G.E. Eide, P.S. Bakke, Remission of respiratory symptoms by smoking and occupational exposure in a cohort study, Eur. Respir. J. 23 (2004) 589–594. http://ovidsp.ovid.com/ovidweb.cgi?T=JS&PAGE=reference&D=med5&NEWS=N&AN=15083759 NS  -. | Trial registration |
| K.J. Donham, J.A. Merchant, D. Lassise, W.J. Popendorf, L.F. Burmeister, Preventing respiratory disease in swine confinement workers: Intervention through applied epidemiology, education, and consultation, Am. J. Ind. Med. 18 (1990) 241–261. http://ovidsp.ovid.com/ovidweb.cgi?T=JS&PAGE=reference&D=emed4&NEWS=N&AN=20307542 NS  -. | Duration of cough not defined |
| H.-Y. Deng, W. Luo, M. Zhang, J.-X. Xie, Z.-Y. Fang, K.-F. Lai, Initial empirical treatment based on clinical feature of chronic cough, Clin. Respir. J. 10 (2016) 622–630. https://doi.org/https://dx.doi.org/10.1111/crj.12270. | Duration of cough not defined |
| E. Daynes, N.J. Greening, T.C. Harvey-Dunstan, S.J. Singh, High-frequency airway oscillating device for respiratory muscle training in subjects with copd, Respir. Care. 63 (2018) 584‐. https://doi.org/10.4187/respcare.05837. | Trial registration |
| D. Damaraju, T. Steiner, J. Wade, K. Gin, J.M. FitzGerald, CLINICAL PROBLEM-SOLVING. A Surprising Cause of Chronic Cough, N. Engl. J. Med. 373 (2015) 561–566. https://doi.org/https://dx.doi.org/10.1056/NEJMcps1303787. | Duration of cough not defined |
| S.E. Daly, K.M. Sundar, W. Dunaway, D. Flinders, Retrospective study of approach and management to chronic cough: Empiricism in therapeutic approach and association with sleep apnea, Chest. 136 (2009). http://meeting.chestpubs.org/cgi/content/abstract/136/4/34S-hhttp://ovidsp.ovid.com/ovidweb.cgi?T=JS&PAGE=reference&D=emed11&NEWS=N&AN=70203887 NS  -. | Cough augmentation |
| M. Dabrowska, E.M. Grabczak, M. Arcimowicz, A. Domeracka-Kolodziej, J. Domagala-Kulawik, R. Krenke, R. Chazan, Chronic cough-assessment of treatment efficacy based on two questionnaires, Allergy Eur. J. Allergy Clin. Immunol. 70 (2015) 648–649. https://doi.org/http://dx.doi.org/10.1111/all.12724. | Duration of cough not defined |
| N.J. Cox, J.C. Hendricks, R.A. Binkhorst, C.L. van Herwaarden, A pulmonary rehabilitation program for patients with asthma and mild chronic obstructive pulmonary diseases (COPD), Lung. 171 (1993) 235‐244. https://doi.org/10.1007/bf00203723. | Cough augmentation |
| D.E. Cowen, Allergy of the respiratory tract: a comprehensive approach to treatment, Otolaryngol. Clin. North Am. 4 (1971) 465–477. http://ovidsp.ovid.com/ovidweb.cgi?T=JS&PAGE=reference&D=med1&NEWS=N&AN=4106822 NS  -. | Duration of cough not defined |
| K.J. Coughlin, R. Hruska, J. Masek, Cough-variant asthma: responsive to integrative management and postural restoration, Explore (NY). 1 (2005) 377–379. http://ovidsp.ovid.com/ovidweb.cgi?T=JS&PAGE=reference&D=med6&NEWS=N&AN=16781569 NS  -. | No intervention |
| R. Choate, C.B. Pasquale, N.A. Parada, V. Prieto-Centurion, B.P. Yawn, Promis-29 scores associated with longitudinal changes in cough and phlegm severity in patients with copd within the chronic obstructive pulmonary disease patient-powered research network (copd pprn), Am. J. Respir. Crit. Care Med. 199 (2019). https://www.atsjournals.org/doi/abs/10.1164/ajrccm-conference.2019.199.1_MeetingAbstracts.A5643http://ovidsp.ovid.com/ovidweb.cgi?T=JS&PAGE=reference&D=emexa&NEWS=N&AN=630353470 NS  -. | No chronic cough (defined as >=8weeks) |
| ChiCtr, Effect of Continuous Nursing Based on IKAP theory on the Quality of Life in Patients with Chronic Obstructive Pulmonary Diseaseâ€”â€”Randomized Controlled Study, Http://Www.Who.Int/Trialsearch/Trial2.Aspx?TrialID=ChiCTR1900024383. (2019). https://www.cochranelibrary.com/central/doi/10.1002/central/CN-01975061/full NS  -. | No chronic cough (defined as >=8weeks) |
| C.T. Chi, The effects of the respiratory movement control training system on respiratory function in patients with pulmonary diseases, Http://Www.Who.Int/Trialsearch/Trial2.Aspx?TrialID=ChiCTR-TRC-13003400. (2013). https://www.cochranelibrary.com/central/doi/10.1002/central/CN-01822125/full NS  -. | No chronic cough (defined as >=8weeks) |
| J. Chen, S. Chen, Z. Chen, TCM treatment of interstitial pneumonia with chronic cough - A case report, J. Trad. Chin. Med. 23 (2003) 170–171. https://www.scopus.com/inward/record.uri?eid=2-s2.0-0642305055&partnerID=40&md5=83258e38c8cd5486dd19e6483379cf13 NS  -. | Duration of cough not defined |
| J.G. Clarke, S.A. Martin, R.A. Martin, L.A.R. Stein, J.J. van den Berg, D.R. Parker, A.R. McGovern, M.B. Roberts, B.C. Bock, Changes in smoking-related symptoms during enforced abstinence of incarceration, J. Health Care Poor Underserved. 26 (2015) 106–118. https://doi.org/https://dx.doi.org/10.1353/hpu.2015.0014. | Duration of cough not defined |
| A. Brzecka, M. Pawelec-Winiarz, P. Piesiak, E. Nowak, R. Jankowska, Suppression of chronic nocturnal cough during continuous positive airway pressure (CPAP) treatment in a patient with asthma and obstructive sleep apnea syndrome, Pneumonol. Alergol. Pol. 79 (2011) 121–126. http://www.pneumonologia.viamedica.pl/en/darmowy_pdf.phtml?indeks=34&indeks_art=496http://ovidsp.ovid.com/ovidweb.cgi?T=JS&PAGE=reference&D=emed12&NEWS=N&AN=361388182 NS  -. | Duration of cough not defined |
| R. Bonnet, R. Jorres, R. Downey, H. Hein, H. Magnussen, Intractable cough associated with the supine body position. Effective therapy with nasal CPAP, Chest. 108 (1995) 581–585. http://ovidsp.ovid.com/ovidweb.cgi?T=JS&PAGE=reference&D=med3&NEWS=N&AN=7634907 NS  -. | Duration of cough not defined |
| H.W. Bonekat, R.M. Miles, B.A. Staats, Smoking and cough syncope: follow-up in 45 cases, Int. J. Addict. 22 (1987) 413–419. http://ovidsp.ovid.com/ovidweb.cgi?T=JS&PAGE=reference&D=med2&NEWS=N&AN=3596855 NS  -. | Intervention not reported |
| B. Celli, D. Halpin, R. Hepburn, N. Byrne, E.T. Keating, M. Goldman, Symptoms are an important outcome in chronic obstructive pulmonary disease clinical trials: results of a 3-month comparative study using the Breathlessness, Cough and Sputum Scale (BCSS), Respir. Med. 97 Suppl A (2003) S35‐43. https://www.cochranelibrary.com/central/doi/10.1002/central/CN-00422593/full NS  -. | Trial registration |
| Y. Cao, S.H. Lin, D. Zhu, F. Xu, Z.H. Chen, H.H. Shen, W. Li, WeChat Public Account Use Improves Clinical Control of Cough-Variant Asthma: a Randomized Controlled Trial, Med. Sci. Monit. 24 (2018) 1524‐1532. https://doi.org/10.12659/msm.907284. | No intervention |
| S. Campbell, R. Stacey, J. Haines, S. Lillie, S.J. Fowler, A. Vyas, Multidisciplinary treatment for vocal Cord dysfunction: A case of improved symptom presentation and management, Am. J. Respir. Crit. Care Med. 185 (2012). http://www.atsjournals.org/doi/pdf/10.1164/ajrccm-conference.2012.185.1_MeetingAbstracts.A4199http://ovidsp.ovid.com/ovidweb.cgi?T=JS&PAGE=reference&D=emed13&NEWS=N&AN=71988834 NS  -. | Duration of cough not defined |
| A. Bodenes, M. Andre, J.D. Dewitte, J.J. Quiot, G. Potard, P. Mialon, P. Gales, C. Leroyer, An occupational vocal dysfunction syndrome?, Arch. Des Mal. Prof. Med. Du Trav. 63 (2002) 87–90. http://ovidsp.ovid.com/ovidweb.cgi?T=JS&PAGE=reference&D=emed7&NEWS=N&AN=34556020 NS  -. | Pharmacological intervention |
| F.B. Blager, M.L. Gay, R.P. Wood, Voice therapy techniques adapted to treatment of habit cough: a pilot study, J. Commun. Disord. 21 (1988) 393–400. http://ovidsp.ovid.com/ovidweb.cgi?T=JS&PAGE=reference&D=med3&NEWS=N&AN=3183084 NS  -. | Pharmacological intervention |
| S.S. Birring, B. Prudon, A.J. Carr, S.J. Singh, M.D.L. Morgan, I.D. Pavord, Development of a symptom specific health status measure for patients with chronic cough: Leicester Cough Questionnaire (LCQ), Thorax. 58 (2003) 339–343. http://ovidsp.ovid.com/ovidweb.cgi?T=JS&PAGE=reference&D=med5&NEWS=N&AN=12668799 NS  -. | Duration of cough not defined |
| M.S. Bhatia, R. Chandra, L. Vaid, Psychogenic cough: a profile of 32 cases, Int. J. Psychiatry Med. 32 (2002) 353–360. http://ovidsp.ovid.com/ovidweb.cgi?T=JS&PAGE=reference&D=med4&NEWS=N&AN=12779185 NS  -. | Secondary source |
| M. Bernabeu Lledo, M.A. Atin Arratibel, M. Martinez Tardido, M. Gonzalez Cifuentes, R. Fuertes Conejo, Efficacy of respiratory physiotherapy combined with ventilation percussive intrapulmonary (VPI) in stable adult, Ann. Phys. Rehabil. Med. 57 (2014) e326. https://doi.org/10.1016/j.rehab.2014.03.1196. | Wrong language |
| K. Chan, G. Cossa, L. Laks, S. Birring, A. Ing, Impact on objective cough severity by continuous positive airway pressure (CPAP) in subjects with chronic cough and obstructive sleep apnoea-a randomized controlled trial, Eur. Respir. J. 38 (2011). http://erj.ersjournals.com/content/38/Suppl_55/p484http://ovidsp.ovid.com/ovidweb.cgi?T=JS&PAGE=reference&D=emed12&NEWS=N&AN=72122710 NS  -. | Duration of cough not defined |
| K. Chan, S. Birring, G. Cossa, L. Laks, P. Rogers, A. Ing, Impact of continuous positive airway pressure (CPAP) on chronic cough in obstructive sleep apnoea (OSA)-a randomized controlled trial, J. Sleep Res. 23 (2014) 153. https://doi.org/http://dx.doi.org/10.1111/jsr.12213. | Duration of cough not defined |
| K. Barraclough, Chronic cough in adults, BMJ. 338 (2009) b1218. https://doi.org/https://dx.doi.org/10.1136/bmj.b1218. | Duration of cough not defined |
| T.R. Baker, M. Oscherwitz, R. Corlin, T. Jarboe, J. Teisch, M.Z. Nichaman, Screening and treatment program for mild chronic obstructive pulmonary disease, JAMA. 214 (1970) 1448–1455. http://ovidsp.ovid.com/ovidweb.cgi?T=JS&PAGE=reference&D=med1&NEWS=N&AN=5536345 NS  -. | Duration of cough not defined |
| C. Badr, M.R. Elkins, E.R. Ellis, The effect of body position on maximal expiratory pressure and flow, Aust. J. Physiother. 48 (2002) 95‐102. https://doi.org/10.1016/s0004-9514(14)60203-8. | Duration of cough not defined |
| S. Asker, M. Asker, A case report of Obstructive Sleep Apnea Syndrome admitted to the hospital with chronic cough, Respir. Case Reports. 2 (2013) 154–157. https://doi.org/http://dx.doi.org/10.5505/respircase.2013.09709. | Duration of cough not defined |
| O.O. Adeyeye, Y.A. Kuyinu, T.R. Bamisile, C.I. Oghama, A preliminary assessment of nurses’ asthma education needs and the effect of a training programme in an urban tertiary healthcare facility, African J. Respir. Med. 10 (2015) 13–17. http://www.africanjournalofrespiratorymedicine.com/articles/march_2015/AJRM-111 (Adeyeye).pdfhttp://ovidsp.ovid.com/ovidweb.cgi?T=JS&PAGE=reference&D=emed16&NEWS=N&AN=604772126 NS  -. | Duration of cough not defined |
| M. Adessa, R. Xiao, A.J. Bowen, P.C. Bryson, Effects of behavioral cough suppression therapy in the treatment of chronic refractory cough, Otolaryngol. - Head Neck Surg. (United States). 159 (2018) P110. https://doi.org/http://dx.doi.org/10.1177/0194599818785627e. | Duration of cough not defined |
| B.O. Adefuye, O.O. Odusan, O.J. Ogunkoya, Sarcoidosis in a male nigerian diabetic patient, Am. J. Respir. Crit. Care Med. 181 (2010). http://ajrccm.atsjournals.org/cgi/reprint/181/1_MeetingAbstracts/A4524?sid=3bc2b9c9-3093-4521-a7ed-d2a0457f612ahttp://ovidsp.ovid.com/ovidweb.cgi?T=JS&PAGE=reference&D=emed11&NEWS=N&AN=70841974 NS  -. | Duration of cough not defined |
| A. Michalowski, A. Haines, N. Shaparin, K. Gritsenko, A.D. Kaye, E.M. Cornett, M.Z.A.O.-L. Lerner  Michael Z.; ORCID: http://orcid.org/0000-0002-8715-4533, Transcutaneous Electrical Nerve Stimulation as a Treatment for Neuropathic Cough: A Tolerability and Feasibility Study, Neurol. Ther. (2021). https://doi.org/http://dx.doi.org/10.1007/s40120-021-00255-2. | Wrong study design |
| K.M. Sundar, A.M. Willis, S. Smith, N. Hu, J.P. Kitt, S.S. Birring, A Randomized, Controlled, Pilot Study of CPAP for Patients with Chronic Cough and  Obstructive Sleep Apnea., Lung. 198 (2020) 449–457. https://doi.org/10.1007/s00408-020-00354-1. | Abstract in conference proceedings |
| T. Murry, T. Virtue, J. Datema, C. Liem, B. Crawley, P. Krishna, Expiratory muscle strength training as treatment for chronic cough, Lung. 198 (2020) 39. https://doi.org/http://dx.doi.org/10.1007/s00408-020-00328-3. | Repeat |
| A.E. Vertigan, S.L. Kapela, N.M. Ryan, S.S. Birring, P. McElduff, P.G. Gibson, Pregabalin and Speech Pathology Combination Therapy for Refractory Chronic Cough: a Randomized Controlled Trial, Chest. 149 (2016) 639‐648. https://doi.org/10.1378/chest.15-1271. | Pharmacological intervention |
